# Description of two new sympatric species of the genus *Leptolalax* (Anura: Megophryidae) from western Yunnan of China

**DOI:** 10.7717/peerj.4586

**Published:** 2018-04-10

**Authors:** Jian-Huan Yang, Zhao-Chi Zeng, Ying-Yong Wang

**Affiliations:** 1 Kadoorie Conservation China, Kadoorie Farm and Botanic Garden, Hong Kong, China; 2 State Key Laboratory of Biocontrol/The Museum of Biology, School of Life Sciences, Sun Yat-sen University, Guangzhou, Guangdong, China

**Keywords:** Amphibians, Leaf litter toads, Taxonomy, Bioacoustics, Molecular, Yingjiang county

## Abstract

The Asian leaf litter toads of the genus *Leptolalax* represent a highly diverse species group and currently contain 53 recognized species. During herpetological surveys in Yingjiang County, western Yunnan of China, we collected series of *Leptolalax* specimens from an isolated small fragment of montane evergreen forest. Subsequent study based on acoustic, morphological and molecular data reveals that there were three different species among the specimens sampled: while one of them belongs to *Leptolalax ventripunctataus*, the other two species represent unknown taxa and are described herein: *Leptolalax purpurus* **sp. nov.** and *Leptolalax yingjiangensis* **sp. nov**. The two new species can be distinguished from other congeners by the molecular divergences, acoustic data, and by a combination of morphological characters including: body size, dorsal and ventral patterns, dorsal skin texture, sizes of pectoral and femoral glands, degree of webbing and fringing on the toes and fingers, dorsum coloration and iris coloration in life. Our results further reveal that species diversity of the genus *Leptolalax* still remains highly underestimated and warrants further attention.

## Introduction

The Asian leaf litter toads of the genus *Leptolalax* Dubois, 1983 are widely distributed from southern China west to northeastern India and Myanmar, through mainland Southeast Asia to the island of Borneo. Fifty-three nominal species within the genus are recognized to date, with more than half described in the past ten years (Frost, 2017), in particular from Indochina and southern China (Yang et al., 2016; Rowley et al., 2016; Rowley, Dau & Cao, 2017; Yuan et al., 2017). Currently, two subgenera are recognized in the genus: the nominal subgenus *Leptolalax* is distributed south of the Isthmus of Kra, while the subgenus *Lalos* Dubois, Grosjean, Ohler, Adler & Zhao is distributed north of the Isthmus of Kra (Delorme et al., 2006; Dubois et al., 2010; Ohler et al., 2011). However, due to the limited mtDNA datasets of the genus, there is no firm phylogenetic support for this division (Poyarkov et al., 2015; Matsui et al., 2017). In this paper, we followed most recent papers to retain the recognition of the subgenus *Lalos* until a more well-supported phylogenetic study is available (Poyarkov et al., 2015; Yang et al., 2016; Rowley, Dau & Cao, 2017; Yuan et al., 2017).

In between May 2016 and June 2017, during field biodiversity surveys in an isolated patch of mature montane evergreen forest in Yingjiang County, Yunnan Province of China, we discovered three different species of the genus *Leptolalax* co-occurring in this small forest fragment. These three different species can be easily separated from each other in appearance and by male calls in the wild. Subsequent study incorporating acoustic, morphological and molecular data revealed that one of them can be allocated to *L. ventripuntactus* Fei, Ye & Li, while the other two species differ from each other and all other recognized congeners, and represent two new species of *Leptolalax* which we describe herein.

## Materials and Methods

**Figure 1:**
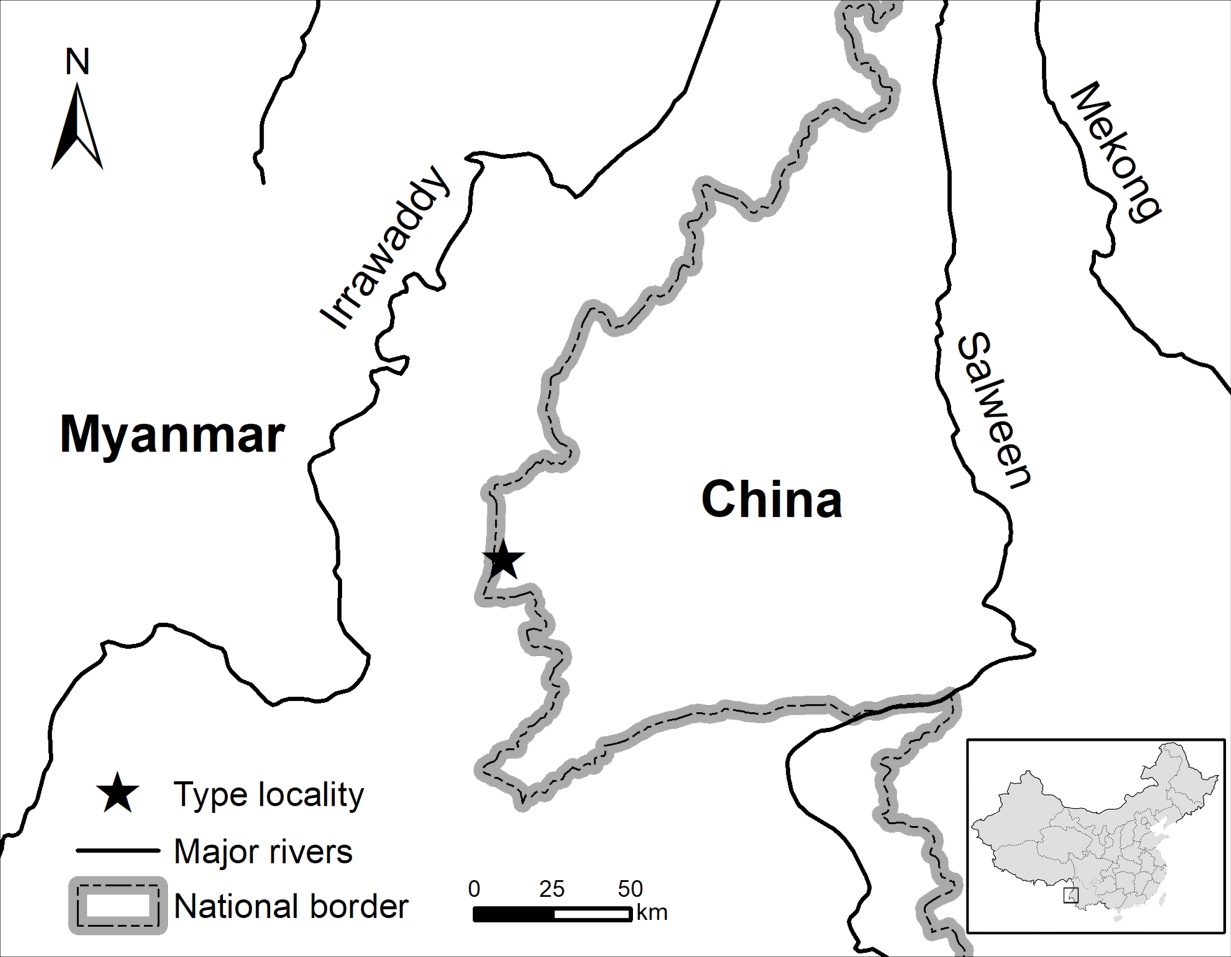
Map showing the type locality of the new species. Map showing the type locality of *Leptolalax purpurus* **sp. nov.**and *Leptolalax yingjiangensis* **sp. nov.**in Tongbiguan Town, Yingjiang County of Yunnan Province, China.

**Sampling.** A total of 15 specimens were collected during fieldwork in Yingjiang County, Yunnan Province between May 2016 and June 2017. All specimens were fixed and preserved in 80% ethanol and deposited at The Museum of Biology, Sun Yat-sen University (SYS). The geographic position of the surveyed locality is shown in Fig. 1. All the animal operations were approved by the Institutional Ethical Committee of Animal Experimentation of Sun Yat-sen University (2005DKA21403-JK).

**Acoustic analyses.** The advertisement call of the three *Leptolalax* species from Yingjiang, Yunnan, China were recorded in the field, using a TASCAM DR-40 digital sound recorder held within 50 cm of the calling individuals. The sound files in wave format were resampled at 48 kHz with sampling depth 24 bits. The sonograms and waveforms were generated by Raven Pro 1.5 software (The Cornell Lab of Ornithology, available from http://www.birds.cornell.edu/raven) with Fast Fourier samples 512 points and overlap 50%, from which all parameters and characters were measured as following Rowley et al. (2012). Comparative advertisement call characters for congeners in the subgenus *Lalos* species were taken from references, with advertisement calls known for 18 out of the 38 known species of in the subgenus *Lalos* (Matsui, 2006; Rowley & Cao, 2009; Rowley et al., 2010a; Rowley et al., 2010b; Rowley et al., 2010c; Rowley et al., 2011; Rowley et al., 2012; Rowley, Dau & Nguyen, 2013; Rowley et al., 2015a; Rowley et al., 2016; Rowley et al., 2017; Poyarkov et al., 2015; Rowley, Dau & Cao, 2017).

**DNA Extraction, PCR and sequencing.** DNA was extracted from muscle tissue using a DNA extraction kit from Tiangen Biotech (Beijing, China) Co., Ltd. The mitochondrial gene 16S ribosomal RNA gene (16S rRNA) from all samples were sequenced using primer pairs L3975 (5′-CGCCTGTTTACCAAAAACAT-3′) and H4551 (5′-CCGGTCTGAACTCAGATCACGT-3′) (Simon et al., 1994). PCR amplifications were performed in a 20 µl reaction volume with the following cycling conditions: an initial denaturing step at 95 °C for 4 min; 35 cycles of denaturing at 94 °C for 40 s, annealing at 53 °C for 40 s and extending at 72 °C for 1 min, and a final extending step of 72 °C for 10 min. PCR products were purified with spin columns. The purified products were sequenced with both forward and reverse primers using BigDye Terminator Cycle Sequencing Kit according to the guidelines of the manufacturer. The products were sequenced on an ABI Prism 3730 automated DNA sequencer in Shanghai Majorbio Bio-pharm Technology Co., Ltd. All sequences have been deposited in GenBank (Table 1).

**Table 1:** Samples and sequence information. Samples and sequences of the mitochondrial 16S rRNA gene used in the phylogenetic analysis in this study.

	Species	Locality	Voucher no.	GenBank no.
1	*L. purpurus* **sp. nov.**	China, Yunnan Province, Yingjiang	SYS a006530	MG520354
2	*L. purpurus* **sp. nov.**	China, Yunnan Province, Yingjiang	SYS a006531	MG520355
3	*L. yingjiangensis* **sp. nov.**	China, Yunnan Province, Yingjiang	SYS a006533	MG520350
4	*L. yingjiangensis* **sp. nov.**	China, Yunnan Province, Yingjiang	SYS a006532	MG520351
5	*L. yingjiangensis* **sp. nov.**	China, Yunnan Province, Yingjiang	SYS a006534	MG520356
6	*L. yingjiangensis* **sp. nov.**	China, Yunnan Province, Yingjiang	SYS a006535	MG520357
7	*L. yingjiangensis* **sp. nov.**	China, Yunnan Province, Yingjiang	SYS a006536	MG520358
8	*L. yingjiangensis* **sp. nov.**	China, Yunnan Province, Yingjiang	SYS a006537	MG520359
9	*L. aereus*	Vietnam, Quang Binh Province	RH60165	JN848437
10	*L. applebyi*	Vietnam, Quang Nam Province	AMS R171703	HM133597
11	*L. arayai*	Malaysia, Borneo	BORNEENSIS 22931	AB847558
12	*L. ardens*	Vietnam, Gia Lai Province	VNMN 04707	KR018109
13	*L. bidoupensis*	Vietnam, Lam Dong Province	AMS R173134	HQ902881
14	*L. bourreti*	Vietnam, Lao Cai Province	AMS R177673	KR018124
15	*L. dringi*	Malaysia, Borneo	KUHE:55610	AB847553
16	*L. eos*	Laos, Phongsaly Province	MNHN:2004.0278	JN848450
17	*L. firthi*	Vietnam, Kon Tum Province	AMS R176524	JQ739206
18	*L. fritinniens*	Malaysia, Borneo	KUHE 55371	AB847557
19	*L. fuliginosus*	Thailand	KUHE:20172	LC201985
20	*L. gracilus*	Malaysia, Borneo	KUHE 55624	AB847560
21	*L. hamidi*	Malaysia, Borneo	KUHE 17545	AB969286
22	*L. heteropus*	Malaysia, Peninsular	KUHE 15487	AB530453
23	*L. isos*	Vietnam, Gia Lai Province	VNMN A 2015.4	KT824769
24	*L. kalonensis*	Vietnam, Binh Thuan Province	IEBR A.2014.15	KR018114
25	*L. khasiorum*	India, Meghalaya	SDBDU 2009.329	KY022303
26	*L. laui*	China, Shenzhen	SYS A002057	KM014546
27	*L. liui*	China, Jiangxi Province	SYS A001620	KM014549
28	*L. maculosus*	Vietnam, Ninh Thuañ Province	ZFMK 96600	KR018120
29	*L. maoershanensis*	China, Guangxi Province	KIZ019385	KY986930
30	*L. marmoratus*	Malaysia, Borneo	KUHE 53227	AB969289
31	*L. maurus*	Malaysia, Borneo	SP 21450	AB847559
32	*L. melicus*	Cambodia, Ratanakiri Province	MVZ 258198	HM133600
33	*L. minimus*	Thailand, Chiang Mai Province	–	JN848369
34	*L. nyx*	Vietnam, Ha Giang Province	AMNH A163810	DQ283381
35	*L. oshanensis*	China, Sichuan Province	SYS A001830	KM014810
36	*L. pallidus*	Vietnam, Lam Dong Province	UNS00510	KR018112
37	*L. petrops*	Vietnam, Tuyen Quang Province	VNMN 2016 A.06	KY459998
38	*L. pictus*	Malaysia, Borneo	UNIMAS 8705	KJ831295
39	*L. pluvialis*	Vietnam, Lao Cai Province	MNHN:1999.5675	JN848391
40	*L. puhoatensis*	Vietnam, Nghe An Province	VNMN 2016 A.22	KY849586
41	*L. pyrrhops*	Vietnam, Lam Dong Province	ZMMU A-5208	KP017575
42	*L. sabahmontanus*	Malaysia, Borneo	BORNEENSIS 12632	AB847551
43	*L. tadungensis*	Vietnam, Dak Nong Province	UNS00515	KR018121
44	*L. tengchongensis*	China, Yunnan Province, Tengchong	SYS a004600	KU589210
45	*L. ventripunctatus*	China, Yunnan Province, Xishuangbanan	SYS a004539	MG520361
46	*L. ventripunctatus*	China, Yunnan Province, Yingjiang	KFBG 14423	MG520352
47	*L. ventripunctatus*	China, Yunnan Province, Yingjiang	KFBG 14509	MG520353
48	*L. ventripunctatus*	China, Yunnan Province, Yingjiang	KFBG 14531	MG520360
49	*L. zhangyangpingi*	Thailand, Chiang Mai Province	–	JX069979
50	*Leptobrachium* cf. *chapaense*	Vietnam, Lao Cai Province	AMS R171623	KR018126
51	*Xenophrys major*	Vietnam, Kon Tum Province	AMS R173870	KY476333

**Phylogenetic analyses.** In addition to the newly collected specimens, sequences of all species of the genus *Leptolalax* for which homologous sequences of 16S rRNA were available (38 of the 53 species) from the Genbank were included in the genetic analyses (Table 1). We used *Leptobrachium chapaense* (Bourret) and *Xenophrys major* (Boulenger) as outgroups. Sequence alignments were first conducted using Clustal X 2.0 (Thompson, Higgins & Gibson, 1994), with default parameters and the alignment being checked and manually revised, if necessary. The data were tested in jmodeltest v2.1.2 with Akaike and Bayesian information criteria, resulting the best-fitting nucleotide substitution models are GTR + I + G. Sequence data were analyzed using maximum likelihood (ML) implemented in RaxmlGUI 1.3 (Silvestro & Michalak, 2012), and Bayesian inference (BI) using MrBayes 3.12 (Ronquist & Huelsenbeck, 2003). The phylogenetic tree was constructed using ML and BI methods. For ML analysis, the bootstrap consensus tree inferred from 1,000 replicates was used to represent the evolutionary history of the taxa analyzed. Branches corresponding to partitions reproduced in less than 70% of bootstrap replicates were collapsed (Hillis & Bull, 1993). For BI analysis, two independent runs with four Markov Chain Monte Carlo simulations were performed for ten million iterations and sampled every 1,000th iteration. The first 25% of samples were discarded as burn-in. Convergence of the Markov Chain Monte Carlo simulations was assessed using Tracer v.1.4 (http://tree.bio.ed.ac.uk/software/tracer/). Pairwise distances based on 16S rRNA were calculated in MEGA 6.06 using the uncorrected *p*-distance model (Tamura et al., 2013).

**Morphological characters.** Measurements followed Fei et al. (2009) and Rowley, Dau & Nguyen (2013), and were taken with digital callipers to the nearest 0.1 mm: snout-vent length (SVL); head length from tip of the snout to rear of jaws (HDL); head width at commissure of jaws (HDW); snout length from tip of the snout to anterior corner of eye (SNT); diameter of exposed portion of eyeball (EYE); interorbital distance (IOD); internasal distance (IND); upper eyelid width measured as greatest width of the upper eyelid (UEW); nostril-eyelid length (NEL); nostril-snout length (NSL); horizontal diameter of tympanum (TMP); distance from anterior edge of tympanum to posterior corner of eye (TEY); tibia length with hindlimb flexed (TIB); manus length from tip of third digit to proximal edge of inner palmar tubercle (ML); length of lower arm and hand (LAHL); pes length from tip of fourth toe to proximal edge of the inner metatarsal tubercle (PL); and hindlimb length from tip of fourth toe to vent (HLL). Sex was determined by direct observation of calling in life and the presence of internal vocal sac openings, and by observation of eggs through translucent belly skin in gravid females. Comparative morphological data of *Leptolalax* species were obtained from the literature (see Appendix S1) and from examination of museum specimens (see Appendix S2). Due to the high likelihood of undiagnosed diversity within the genus, where available, we relied on examination of topotypic material and/or original species descriptions.

**Nomenclatural acts.** The electronic version of this article in Portable Document Format (PDF) will represent a published work according to the International Commission on Zoological Nomenclature (ICZN), and hence the new names contained in the electronic version are effectively published under that Code from the electronic edition alone. This published work and the nomenclatural acts it contains have been registered in ZooBank, the online registration system for the ICZN. The ZooBank LSIDs (Life Science Identifiers) can be resolved and the associated information viewed through any standard web browser by appending the LSID to the prefix http://zoobank.org/. The LSID for this publication is: urn:lsid:zoobank.org:pub:31C06247-A201-42AD-8FF8-B6510530C39D. The online version of this work is archived and available from the following digital repositories: PeerJ, PubMed Central and CLOCKSS.

## Results

### Description of new species

Our detailed morphological study supports the recognition of the two new species of the genus *Leptolalax*, which can be reliably differentiated from each other and all known congeners on the basis of body size, dorsal and ventral patterns, dorsal skin texture, sizes of pectoral and femoral glands, degree of webbing and fringing on the toes and fingers, dorsal coloration and iris coloration in life. These two new taxa are formally described below.

**Table:** 

**Figure 2:**
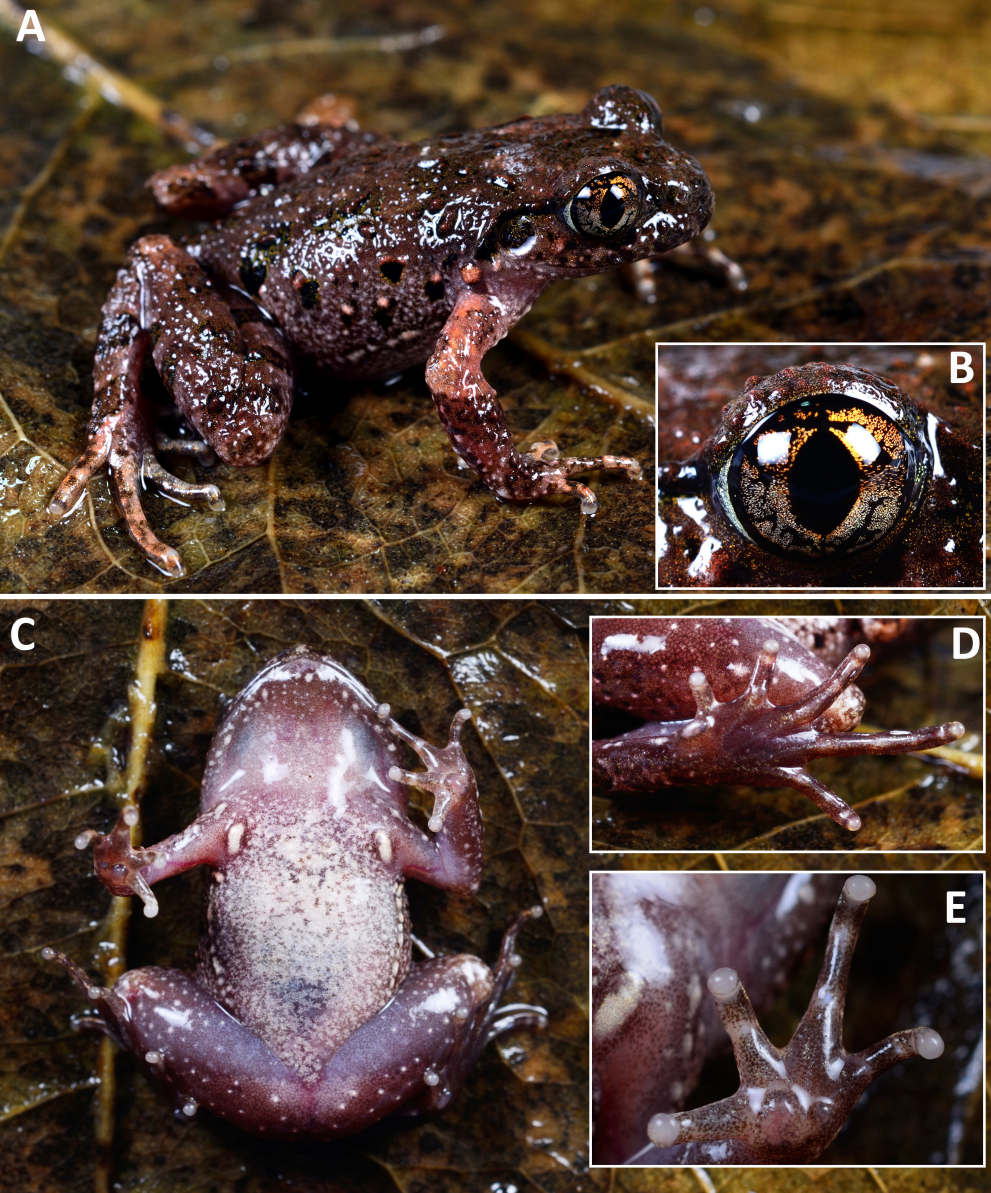
Photos of holotype of* Leptolalax purpurus* sp. nov. Holotype of *Leptolalax purpurus* **sp. nov.** (SYS a006531) in life: (A) dorsolateral view; (B) iris coloration; (C) ventral view; (D) plantar view of the left foot; (D) volar view of the left hand. Photos by JH Yang.

**Figure 3:**
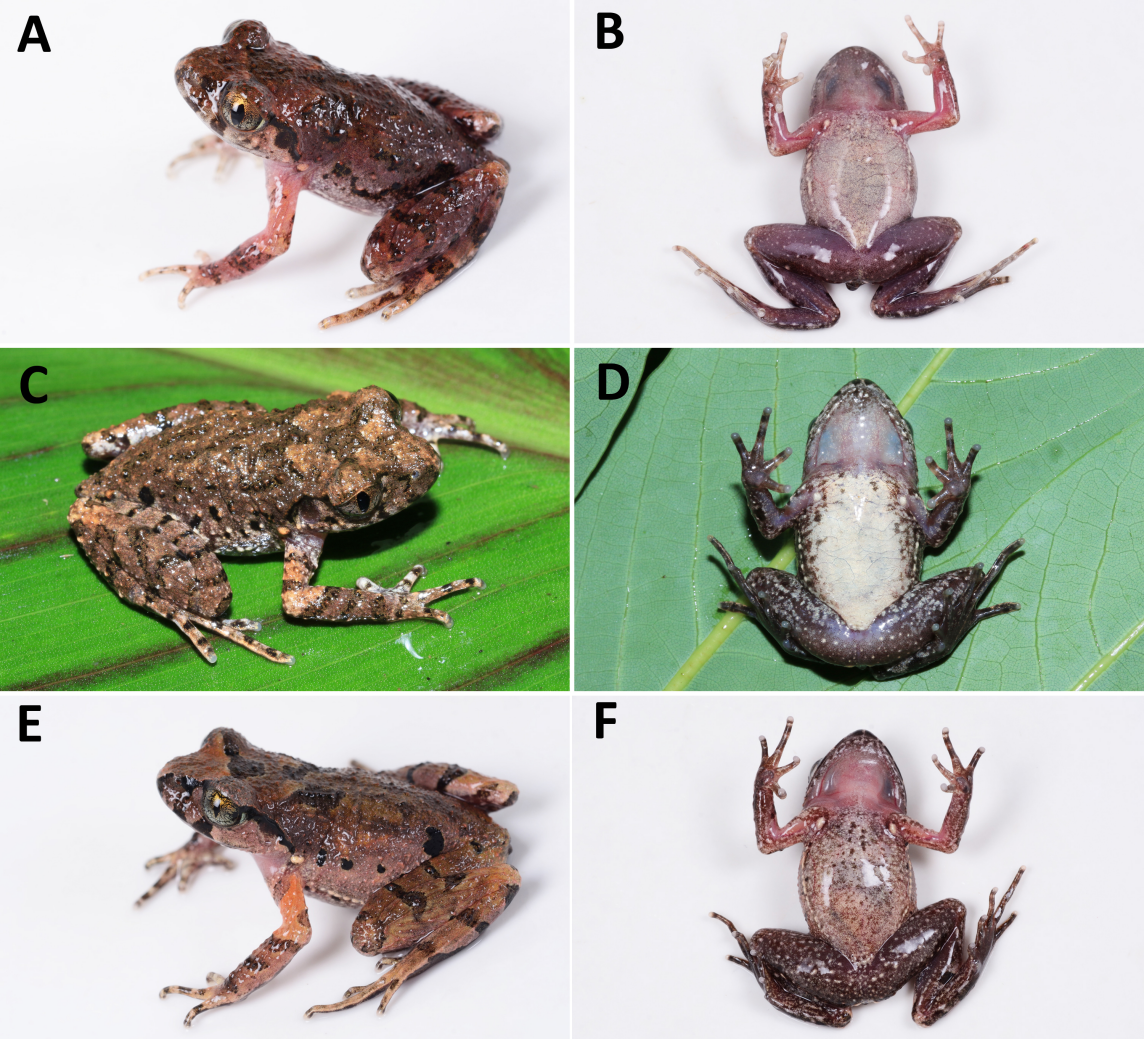
Comparisons of dorsal and ventral color patterns in life of three sympatric* Leptolalax* species. Comparisons of dorsal and ventral color patterns in life of three sympatric* Leptolalax* species in Tongbiguan Town, Yingjiang County of Yunnan Province: (A–B) paratype SYS a006532 of *Leptolalax purpurus* **sp. nov.**; (C–D) paratype SYS a006535 of *Leptolalax yingjiangensis* **sp. nov.**; (E–F) *Leptolalax ventripuntactus*, KFBG 14512. Photos by JH Yang.

**Figure 4:**
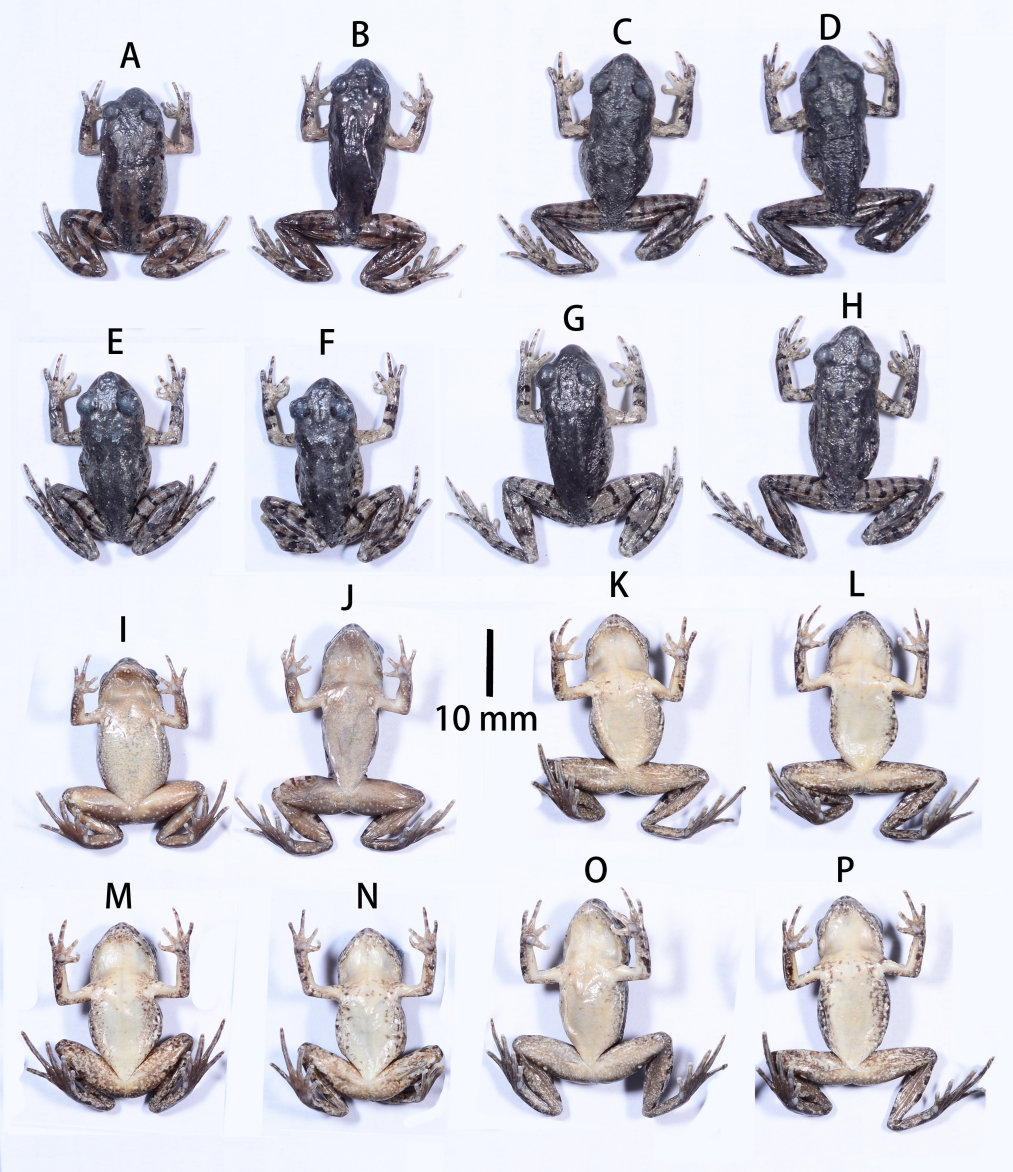
Dorsal and ventral color patterns of type specimens in preservation of the two new *Leptolalax* species. Dorsal and ventral color patterns of type specimens in preservation of the two new *Leptolalax* species in Tongbiguan Town, Yingjiang County of Yunnan Province. *Leptolalax purpurus* **sp. nov.**: SYS a006530 (A & I) and SYS a006531 (B & J); *Leptolalax yingjiangensis* **sp. nov.**: SYS a006532 (C & K), SYS a006533 (D & L), SYS a006534 (E & M), SYS a006535 (F & N), SYS a006536 (G & O), SYS a006537 (H & P). Photos by JH Yang.

***Leptolalax purpurus* sp. nov.**
urn:lsid:zoobank.org:act:40673093-C3A4-4F0C-AD60-C0B517FCEF70
Figs. 2–4; Tables 2–4

**Holotype.** SYS a006531, adult male, collected from Jinzhuzhai Village, Tongbiguan Town, Yingjiang County, Yunnan Province, China (24°37′33.32″N, 97°37′11.91″E, 1,615 m a.s.l.), on 7 December 2016 by J.H. Yang.

**Paratype.** SYS a006530, adult male, same locality as holotype, collected on 20 April 2017 by JH Yang.

**Etymology.** The species epithet, “*purpurus*”, is given as a noun in apposition and means “purple color”, in reference to the purplish dorsum coloration in life of the new species. For the common name, we suggest “Purplish-brown Leaf Litter Toad” (English) and “紫棕掌突蟾” (Chinese).

**Diagnosis.** *Leptolalax purpurus* **sp. nov.** can be distinguished from its congeners by a combination of the following characters: (1) small size (SVL 25.0–27.5 mm in males); (2) dorsal skin shagreened and scattered with fine, round reddish tubercles; (3) tympanum distinctly discernible, almost entirely black; (4) webbing and lateral fringes on fingers absent; (5) toes with rudimentary webbing and wide lateral fringes; (6) pectoral gland larger than tips of fingers and femoral gland; (7) ventrolateral glands distinct; (8) dorsum coloration purplish brown in life; (9) flanks with distinct irregular black spots; (10) black marking/spots on dorsum and flanks mottled with distinct yellow pigmentation in life; (11) ventral side dull white with indistinct grey dusting; (12) relatively short hindlimbs (males TIB/SVL ratio 0.43–0.45 in *purpurus*; males HLL/SVL ratio 1.41–1.48); (13) iris bicolored, upper half orange yellow, lower half sliver white; (14) a call consisting of a single note and a dominant frequency of 4.3–4.5 kHz (at 15 °C).

**Table 2:** Measurement data of the new species. Measurements (in mm) of type specimens of *Leptolalax purpurus* **sp. nov.** and *Leptolalax yingjiangensis* **sp. nov.** Abbreviations defined in text.

	*Leptolalax purpurus* sp. nov.			*Leptolalax yingjiangensis* sp. nov.						
	SYS a006530	SYS a006531	Mean ± SD (*N* = 2)	SYS a006534	SYS a006535	SYS a006536	SYS a006537	SYS a006533	SYS a006532	Mean ± SD (*N* = 6)
Sex	m	m		m	m	m	m	m	m	
SVL	27.5	25.0	26.3 ± 1.77	27.6	27.5	26.2	26.3	25.7	25.7	26.5 ± 0.85
HDL	9.5	9.4	9.5 ± 0.07	10.5	10.4	9.9	10.2	10.2	10.3	10.3 ± 0.21
HDW	8.8	9.2	9.0 ± 0.28	9.9	9.6	9.2	9.6	9.5	9.7	9.6 ± 0.23
SNT	4.1	3.9	4.0 ± 0.14	4.5	4.6	4.3	4.3	4.2	4.2	4.4 ± 0.16
EYE	3.5	3.4	3.5 ± 0.07	4.1	3.9	3.7	3.7	3.8	3.9	3.9 ± 0.15
IOD	3.0	2.7	2.9 ± 0.21	3.1	3.1	3.1	3.5	3.0	3.0	3.1 ± 0.19
TMP	1.8	1.8	1.8 ± 0.00	1.7	1.6	1.7	1.7	1.6	1.8	1.7 ± 0.08
TEY	1.0	1.0	1.0 ± 0.00	1.2	1.1	1.1	1.0	1.1	1.1	1.1 ± 0.06
TIB	11.7	11.3	11.5 ± 0.28	13.1	13.1	12.5	12.6	12.4	12.7	12.7 ± 0.30
ML	11.0	11.3	11.2 ± 0.21	13.3	12.9	12.3	12.6	13.1	12.5	12.8 ± 0.38
PL	11.0	10.9	11.0 ± 0.07	12.8	12.5	11.6	11.5	11.2	11.7	11.9 ± 0.62
LAHL	12.0	11.8	11.9 ± 0.14	14.4	14.2	14.0	14.2	13.6	14.1	14.1 ± 0.27
HLL	38.9	37.1	38.0 ± 1.27	43.7	43.9	41.1	42.5	41.8	42.4	42.6 ± 1.08
LFT	17.3	16.3	16.8 ± 0.71	19.0	19.1	18.3	18.3	18.4	18.8	18.7 ± 0.36
HDL/HDW	1.08	1.02	1.05 ± 0.04	1.06	1.08	1.08	1.06	1.07	1.06	1.07 ± 0.01
HDL/SVL	0.35	0.38	0.36 ± .02	0.38	0.38	0.38	0.39	0.40	0.40	0.39 ± 0.01
EYE/SNT	0.85	0.87	0.86 ± 0.01	0.91	0.85	0.86	0.86	0.90	0.93	0.89 ± 0.03
TMP/EYE	0.51	0.53	0.52 ± 0.01	0.41	0.41	0.46	0.46	0.42	0.46	0.44 ± .02
TIB/SVL	0.43	0.452	0.44 ± 0.02	0.47	0.48	0.48	0.48	0.48	0.49	0.48 ± .01
LAHL/SVL	0.44	0.47	0.45 ± 0.03	0.52	0.52	0.53	0.54	0.53	0.55	0.53 ± 0.01
HLL/SVL	1.41	1.48	1.45 ± .05	1.58	1.60	1.57	1.62	1.63	1.65	1.61 ± 0.03

**Description of holotype.** SYS a006531, adult male, body size small (SVL 25.0 mm), head width (9.2 mm) about equal to head length (9.4 mm); snout slightly protruding, projecting slightly beyond margin of the lower jaw; nostril equidistant between snout and eye; canthus rostralis gently rounded; loreal region slightly concave; interorbital space flat, larger (IOD 2.7 mm) than upper eyelid (2.5 mm in width) and internarial distance (2.6 mm); pineal ocellus absent; pupil vertical; eye diameter smaller than snout length; tympanum distinct, round, diameter (TMP 1.8 mm) smaller than that of the eye (EYE 3.3 mm), and larger than tympanum-eye distance (TEY 1.0 mm); tympanic rim distinctly elevated relative to skin of temporal region; vomerine teeth absent; vocal sac openings slit-like, located posterolaterally on floor of mouth in close proximity to the margins of the mandible; tongue long, wide, with a small shallow notch at posterior tip; supratympanic ridge distinct, extending from eye to supra-axillary gland; a few indistinct tubercles present on supratympanic ridge. Tips of fingers rounded, slightly swollen; relative finger lengths I = II = IV < III; nuptial pad absent; subarticular tubercles absent; a large, round inner palmar tubercle distinctly separated from small, round outer palmar tubercle; finger webbing and dermal fringes absent. Tips of toes similar to that of fingers; relative toe length I <II <V <III <IV; subarticular tubercles absent; distinct dermal ridges present under the 3rd to 5th toes; large, oval inner metatarsal tubercle present, outer metatarsal tubercle absent; toe webbing rudimentary; wide lateral fringes present on all toes. Tibia 45% of snout-vent length; tibiotarsal articulation reaches to posterior corner of the eye. Skin on dorsum shagreened and scattered with irregular fine, round tubercles; longitudinal skin folds on dorsum absent; ventral skin smooth; pectoral gland large, elongated oval, 1.8 mm in length, greatly larger than tips of fingers and femoral gland; femoral gland small, round, 0.6 mm in diameter, distinctly smaller than tips of toes, situated on posteroventral surface of thigh, closer to knee than to vent; supra-axillary gland raised, 1.1 mm in diameter; ventrolateral gland distinct as small white dots forming an incomplete line.

**Coloration of holotype in life.** Dorsal surface purplish brown with indistinct darker brown markings, and two indistinct light coppery orange spots on shoulder region (Fig. 2): V-shaped interorbital marking disconnected to the W-shaped marking between axillae; dorsum scattered with indistinct irregular black spots edged with yellow pigmentation; fine, purplish red tubercles on upper eyelids, snout, head, upper lips, dorsal surfaces of body and limbs, those on flanks somewhat whitish; white bar on tip of the snout; upper lip mottled with black blotches; a distinct small coppery orange tubercle present on below posterior corner of eye; a black spot present on loreal region; lower margin of supratympanic ridge distinctly black; tympanum nearly fully black; transverse dark brown cross-bars presents on dorsal surface of limbs; a few distinct large black spots present on flanks from groin to axilla, the one on groin largest; black markings and spots on dorsum, flank and tympanum mottled with distinct yellow pigmentation; a few coppery orange small tubercles present on flanks; elbow and upper arms in distinct coppery orange coloration; fingers and toes with transverse bars. Venter dull white with indistinct grey dusting; throat immaculate pinkish; ventral surfaces of thigh pinkish and sparsely scattered with tiny white dots; margin of lower lip scattered with tiny and small white spots. Supra-axillary gland coppery orange; femoral, pectoral and ventrolateral glands white and distinct. Iris bicolored, upper half orange yellow, lower half sliver white.

**Coloration of holotype in preservative.** Dark brown markings on dorsum and flanks still visible, while transverse cross-bars on limbs become more distinct; ventral surface of body dull white with grey dusting; throat much darker; macroglands on the ventral surfaces still distinct.

**Variations.** The single male paratype SYS a006530 greatly matches overall characters of the holotype (for measurements of the type series see Table 2), except for the grey dusting on venter much denser and forming a nearly immaculate dull grey venter in life (see Figs. 3 and 4).

**Table 3:** Acoustic characters of three sympatric *Leptolalax*. Measurements of advertisement call parameters for three sympatric *Leptolalax* species in Yingjiang County of Yunnan.

	*Leptolalax ventripunctataus*	*Leptolalax purpurus* **sp. nov.**	*Leptolalax yingjiangensis* **sp. nov.**
Voucher	Unvouchered	SYS a006530	Unvouchered
Numbers of calls measured	80	20	35
Call duration (s)	0.065–0.430 0.145 ± 0.086 (*N* = 80)	0.086–0.117 0.103 ± 0.012 (*N* = 20)	0.028–0.042 0.036 ± 0.003 (*N* = 35)
Call repetition rate (calls/s)	3.8	1.1	5.8
Intercall interval (s)	0.031–0.416 0.134 ± 0.054 (*N* = 75)	0.430–1.557 0.927 ± 0.428 (*N* = 16)	0.113–0.174 0.134 ± 0.017 (*N* = 34)
Notes/call	3–17 5.3 ± 3.2 (*N* = 80)	1	1
Note duration (s)	0.012–0.028 0.018 ± 0.003 (*N* = 85)	–	–
Internote interval (s)	0.006–0.030 0.017 ± 0.006 (*N* = 83)	–	–
Note repetition rate (notes/s)	37.1	–	–
Dominant frequency (kHz)	6.1–6.4	4.3–4.5	5.7–5.9
Temperature	15 °C	15 °C	19 °C

**Notes.**

Remark: – represents data are sequentially identical to “Call duration”, “Intercall interval” and “Call repetition rate”.

**Table 4:** Morphological comparison analysis. Selected diagnostic characters for the species in the genus *Leptolalax* occurring north of the Isthmus of Kra (modified from Rowley et al., 2017; Yuan et al., 2017).

Species	Male SVL (mm)	Black spots on flanks	Toes webbing	Fringes on toes	Ventral coloration	Dorsal skin texture
*L. purpurus* **sp. nov.**	25.0–27.5, *n* = 2	Yes	Rudimentary	Wide	Dull white with indistinct grey dusting	Shagreened with small tubercles
*L. yingjiangensis* **sp. nov.**	25.7–27.6, *n* = 6	Yes	Rudimentary	Wide	Creamy white with dark brown flecks on chest and margins	Shagreened with small tubercles
*L. aereus*	25.1–28.9, *n* = 28	No	Rudimentary	Wide	Near immaculate creamy white, brown specking on margins	Finely tuberculate
*L. alpinus*	24.0–26.4, *n* = 10	Yes	Rudimentary	Wide in males	Creamy-white with dark spots	Relatively smooth, some with small warts
*L. applebyi*	19.6–22.3, *n* = 9	Yes	Rudimentary	No	Reddish brown with white speckling	Smooth
*L. ardens*	21.3-24.7, *n* = 16	Yes	No	No	Reddish brown with white speckling	Smooth- finely shagreened
*L. bidoupensis*	18.5–25.4, *n* = 12	Yes	Rudimentary	Weak	Reddish brown with white speckling	Smooth
*L. botsfordi*	29.1–32.6, *n* = 7	No	Rudimentary	Narrow	Reddish brown with white speckling	Shagreened
*L. bourreti*	28.0–36.2	Yes	Rudimentary	Weak	Creamy white	Relatively smooth, some with small warts
*L. croceus*	22.2–27.3, *n* = 16	No	Rudimentary	No	Bright orange	Highly tuberculate
*L. eos*	33.1–34.7, *n* = 6	No	Rudimentary	Wide	Creamy white	Shagreened
*L. firthi*	26.4–29.2, *n* = 21	No	Rudimentary	Wide in males	Creamy white	Shagreened with fine tubercles
*L. fuliginosus*	28.2–30.0, *n* = 4	Yes	Rudimentary	Weak	White with brown dusting	Nearly smooth, few tubercles
*L. isos*	23.7–27.9, *n* = 38	No	Rudimentary	Wide in males	Creamy white with white dusting on margins	Mostly smooth, females more tuberculate
*L. kalonensis*	25.8–30.6, *n* = 16	Yes	No	No	Pale, speckled brown	Smooth
*L. khasiorum*	24.5–27.3, *n* = 4	Yes	Rudimentary	Wide	Creamy white	Isolated, scattered tubercles
*L. lateralis*	26.9–28.3, *n* = 4	Yes	Rudimentary	No	Creamy white	Roughly granular
*L. laui*	24.8–26.7, *n* = 11	Yes	Rudimentary	Wide	Creamy white with dark brown dusting on margins	Round granular tubercles
*L. liui*	23.0–28.7, *n* = 20	Yes	Rudimentary	Wide	Creamy white with dark brown spots on chest and margins	Round granular tubercles with glandular folds
*L. maculosus*	24.2–26.6, *n* = 3	Yes	No	No	Brown, less white speckling	Mostly smooth
*L. maoershanensis*	25.2–30.4, *n* = 8	Yes	Rudimentary	Narrow	Creamy white chest and belly with irregular black spots	Longitudinal folds
*L. melicus*	19.5–22.7, *n* = 8	Yes	Rudimentary	No	Reddish brown with white speckling	Smooth
*L. minimus*	25.7–31.4, *n* = 20	Yes	Rudimentary	No	Creamy white	Smooth
*L. nahangensis*	40.8, *n* = 1	Yes	Rudimentary	No	Creamy white with light specking on throat and chest	Smooth
*L. nokrekensis*	26.0–33.0, *n* = 5	Yes	Rudimentary	unknown	Creamy white	Tubercles and longitudinal folds
*L. nyx*	26.7–32.6, *n* = 7	Yes	Rudimentary	No	Creamy white with white with brown margins	Rounded tubercles
*L. oshanensis*	26.6–30.7, *n* = 10	Yes	No	No	Whitish with no markings or only small, light grey spots	Smooth with few glandular ridges
*L. pallidus*	24.5–27.7, *n* = 8	No	No	No	Reddish brown with white speckling	Tuberculate
*L. pelodytoides*	27.5–32.3	Yes	wide	Narrow	Whitish	Small, smooth warts
*L. petrops*	23.6–27.6, *n* = 21	No	No	Narrow	Immaculate creamy white	Highly tuberculate
*L. pluvialis*	21.3–22.3, *n* = 3	Yes	Rudimentary	No	Dirty white with dark brown marbling	Smooth, flattened tubercles on flanks
*L. puhoatensis*	24.2–28.1, *n* = 8	Yes	Rudimentary	Narrow	Reddish brown with white dusting	Longitudinal skin ridges
*L. pyrrhops*	30.8–34.3, *n* = 7	Yes	Rudimentary	No	Reddish brown with white speckling	Slightly shagreened
*L. sungi*	48.3–52.7, *n* = 3	No or small	Wide	Weak	White	Granular
*L. tadungensis*	23.3–28.2, *n* = 10	Yes	No	No	Reddish brown with white speckling	Smooth
*L. tamdil*	32.3, *n* = 1	Yes	Wide	Wide	White	Weakly tuberculate
*L. tengchongensis*	23.9–26.0, *n* = 5	Yes	Rudimentary	Narrow	White with dark brown blotches	Shagreened with small tubercles
*L. tuberosus*	24.4–29.5, *n* = 16	No	Rudimentary	No	White with small grey spots/streaks	Highly tuberculate
*L. ventripunctatus*	25.5–28.0, *n* = 10	Yes	Rudimentary	No	Chest and belly with dark brown spots	Longitudinal skin ridges
*L. zhangyapingi*	45.8–52.5, *n* = 7	No	Rudimentary	Wide	Creamy-white with white with brown margins	Mostly smooth with distinct tubercles

**Distribution and natural history.** *Leptolalax purpurus* **sp. nov.** is currently only known from its type locality in Tongbiguan Town, Yingjiang County of Yunnan Province, China. The male holotype SYS a006531 was found perching under leaf litter nearby a small clear-water rocky stream (ca. 1–2 m in width and ca. 5–20 cm in depth), flowing through a montane evergreen broadleaf forest, on 7 December 2016, and no calls were detected during the survey. Only a single male individual (paratype SYS a006530) was found calling and perching on top of a small rock along the stream on 20 April 2017, and no more calls and individuals were detected during the survey. No male calls and individuals of the new species were detected during other two night surveys on 5 May 2016 and 10 June 2017.

**Figure 5:**
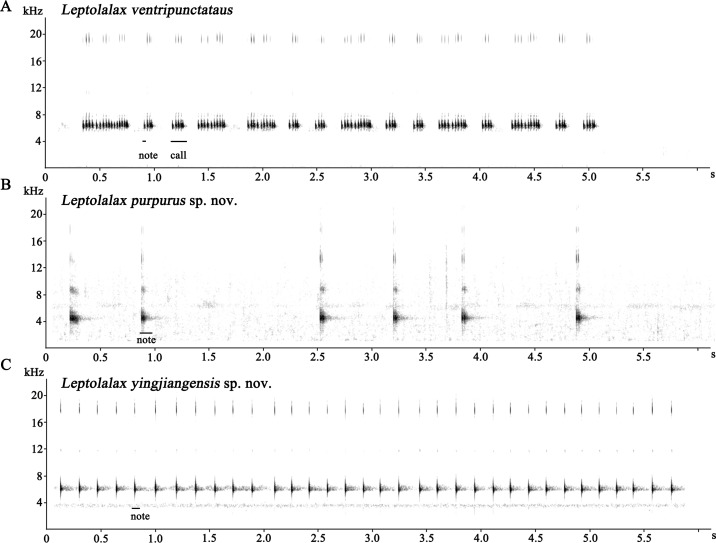
Acoustic analysis of advertisement calls. Advertisement call of the three sympatric * Leptolalax* species from Yingjiang County of Yunnan, China: (A)* Leptolalax ventripuntactus*; (B) *Leptolalax purpurus* **sp. nov.**; (C) *Leptolalax yingjiangensis* **sp. nov.**

**Advertisement calls.** The call of male paratype SYS a006530 of *Leptolalax purpurus* **sp. nov.** was recorded at an ambient temperature of 15 °C on 20 April 2017. The call series contained a numbers of single-note calls with irregular intervals (Fig. 5B). Calls were repeated at a mean rate of 1.1 times per second. Each call was of 0.086–0.117 s duration (mean 0.103 ± 0.012 s) with a peak frequency of 4.3–4.5 kHz. The intercall interval was rather variable and had a relatively long duration of 0.430–1.557 s (mean 0.927 ±0.428 s, *N* = 16) (Table 3).

**Comparison.** By the presence of supra-axillary and ventrolateral glands, *Leptolalax purpurus* **sp. nov.** can be allocated into the subgenus *Lalos*, and distinctly differs from the 15 known species of the subgenus *Leptolalax*, i.e., *L. arayai* Matsui, *L. dringi* Dubois, *L. fritinniens* Dehling & Matsui, *L. gracilis* (Günther), *L. hamidi* Matsui, *L. heteropus* (Boulenger), *L. kajangensis* Grismer, Grismer & Youmans, *L. kecil* Matsui, Belabut, Ahmad & Yong, *L. marmoratus* Matsui, Zainudin & Nishikawa, *L. maurus* Inger, Lakim, Biun & Yambun, *L. melanoleucus* Matsui, *L. pictus* Malkmus, *L. platycephalus* Dehling, *L. sabahmontanus* Matsui, Nishikawa & Yambun and *L. solus* Matsui, all of which occur south of the Isthmus of Kra and lack supra-axillary and ventrolateral glands (Dubois et al., 2010; Dehling & Matsui, 2013; Matsui, Zainudin & Nishikawa, 2014). *Leptolalax purpurus* **sp. nov.** differs from all other species in the subgenus *Lalos* by having purplish brown dorsum in life, pectoral gland greatly larger than femoral gland, black marking/spots on dorsum and flanks mottled with distinct yellow pigmentation, an iris of bicolored coloration, as well as a combination of male body size, presence of black spots on the flank, plus ventral coloration, degree of webbing and fringing on the toes, and dorsal skin texture (See Table  4 for a summarized comparison with all species in the subgenus *Lalos*).

*Leptolalax purpurus* **sp. nov.** differs from the phylogenetically close congener, *L. eos* Ohler, Wollenberg, Grosjean, Hendrix, Vences, Ziegler & Dubois, by having a relatively smaller body size (males SVL 25.0–27.5 mm vs. 33.1–34.7 mm in *eos*), purplish brown above with dark brown markings and spots in life (vs. almost uniformly brown dorsal coloration in *eos*), distinct black spots present on flanks (vs. absent in *eos*), pectoral gland larger than femoral gland (vs. reversed condition in *eos*), ventral surface of body dull white with indistinct grey dusting (vs. immaculate creamy white in *eos*).

**Figure 6:**
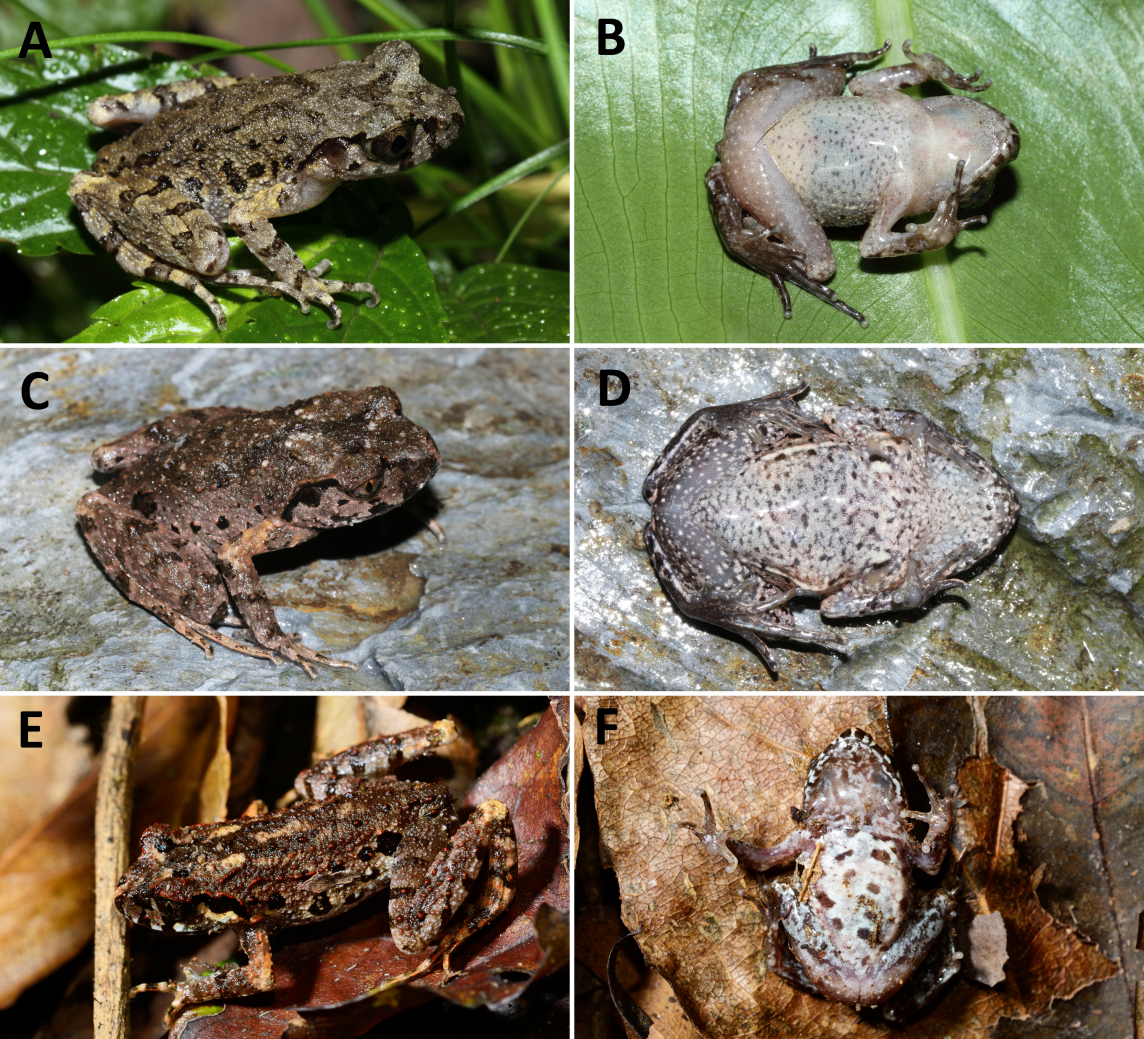
Photos of other three *Leptolalax* species from China. Three other *Leptolalax* species in China: (A–B)* L. oshanensis*, adult male SYS a001829 from its type locality in Mt. Emei, Sichuan Province; (C–D) *L. alpinus*, adult male SYS a003927 from its type locality in Huangcaoling, Jingdong County, Yunnan Province; (E–F) * L. tengchongensis*, male holotype SYS a004600 from Mt. Gaoligongshan, Tengchong, Yunnan Province. Photos by Jian Wang (C & D) and JH Yang (A, B, E & F).

*Leptolalax purpurus* **sp. nov.** differs from the phylogenetically close congener, *L. bourreti* Dubois, by having a relatively smaller body size (males SVL 25.0–27.5 mm vs. 28.0–36.2 mm in *bourreti*), ventral surface dull white with indistinct grey dusting (vs. whitish in *bourreti*), purplish brown above in life (vs. dorsum reddish, greenish or brown in *bourreti*), wide dermal fringes on toes (vs. narrow in *bourreti*), and dermal ridges under toes distinct (vs. poorly distinct in *bourreti*).

*Leptolalax purpurus* **sp. nov.** differs from the phylogenetically close congener, *L. oshanensis* (Liu), by the presence of webbing and dermal fringes on toes (vs. absent in *oshanensis*), purplish brown above in life (vs. reddish or greyish brown in *oshanensis*, see Figs. 6A– 6B), a relatively shorter hindlimb (mean males HLL/SVL ratio 1.45 in *purpurus* **sp. nov.** vs. 1.56 in *oshanensis*), pectoral gland larger than femoral gland (vs. reversed condition in *oshanensis*), femoral gland closer to knee than to vent (vs. reversed condition in *oshanensis*), and the absence of skin folds on dorsum (vs. short skin folds present in *L. oshanensis*).

*Leptolalax purpurus* **sp. nov.** differs from the sympatric *L. ventripunctatus* by having wide dermal fringes on toes (vs. absent or narrow in *ventripunctatus*), absence of longitudinal skin folds on dorsum (vs. present in *ventripunctatus*), dorsal surface purplish brown (vs. brown in *ventripunctatus*, see Fig. 3), black markings and spots on dorsum and flanks mottled with yellow pigmentation (vs. black markings and spots solid black and without such yellow pigmentation in *ventripunctatus*), ventral surface dull white with indistinct grey dusting (vs. distinct small black spots present on venter in *ventripunctatus*, see Fig. 3). See below for the comparison of *Leptolalax purpurus* **sp. nov.** with the other sympatric *Leptolalx* species which is also described as a new species below.

From the rest two known *Leptolalax* species from Yunnan, *Leptolalax purpurus* **sp. nov.** differs from *L. alpinus* Fei, Ye & Li by having tibiotarsal articulation reaches to posterior corner of the eye (vs. reaches anterior corner of the eye in *alpinus*), a relatively shorter hindlimb (mean males HLL/SVL ratio 1.45 in *purpurus* **sp. nov.** vs. 1.55 in *alpinus*), purplish brown above in life (vs. grey brown in *alpinus*), dorsum without white tiny flecks (vs. distinct white tiny flecks present on dorsum in *alpinus*, see Figs. 6C–6D), ventral surface dull white with indistinct grey dusting (vs. distinct dark brown spots/blotches present on belly in *alpinus*); *Leptolalax purpurus* **sp. nov.** differs from *L. tengchongensis* Yang, Wang, Chen & Rao by having wide dermal fringes on toes (vs. narrow in *tengchongensis*), purplish brown above in life (vs. brown in *tengchongensis*), pectoral gland larger than tips of fingers and femoral gland (vs. reversed condition in *tengchongensis*), ventrolateral glands distinct (vs. indistinct in *tengchongensis*); ventral surface dull white with indistinct grey dusting (vs. distinct dark brown blotches present on venter in *tengchongensis* see Figs. 6E–6F), and a bicolored iris (vs. uniform coloration in *tengchongensis*).

*Leptolalax purpurus* **sp. nov.** further differs from *L. pelodytoides* (Boulenger), the only known species from adjoining northeast Myanmar, by having toes webbing rudimentary (vs. wide in *pelodytoides*), wide dermal fringes on toes (vs. narrow in *pelodytoides*), dermal ridges under toes distinct (vs. poorly distinct in *pelodytoides*), ventral surface dull white with indistinct grey dusting (vs. whitish in *pelodytoides*), and the absence of longitudinal skin folds on dorsum (vs. present in *pelodytoides*).

In addition to morphological differences, the advertisement call of *Leptolalax purpurus* **sp. nov.** further differs from all other congeners in the subgenus *Lalos* for which comparable acoustic data are available in having a call consisting invariably of a single note. Of the *Leptolalax* species in the region with known calls, only *L. tuberosus* Inger, Orlov & Darevsky and *L. puhoatensis* Rowley, Dau & Cao were reported having an invariably single-note call, but the call of *Leptolalax purpurus* **sp. nov.** was of a longer duration (86–117 ms at 15 °C vs. 54–78 ms at 22.4–22.5 °C in *tuberosus* and 6–14 ms at 22.3–25.8 °C in *puhoatensis*). In addition, the dominant frequency of 4.3–4.5 kHz (at 15 °C) further distinguishes the call of *Leptolalax purpurus* **sp. nov.** from that of the higher frequency calls of *L. aereus* Rowley, Stuart, Richards, Phimmachak & Sivongxay (6.2–7.9 kHz at 22.4–25.7 °C), *L. firthi* Rowley, Hoang, Dau, Le & Cao (5.4–6.6 kHz at 18.3–21.2 °C), *L. isos* Rowley, Stuart, Neang, Hoang, Dau, Nguyen & Emmett (5.9–6.2 kHz at 22.4–22.8  °C), *L. petrops* Rowley, Dau, Hoang, Le, Cutajar & Nguyen (5.6–6.4 kHz at 24.5–25.3 °C), *L. puhoatensis* (4.9–5.6 kHz at 22.3–25.8 °C) and *L. ventripunctatus* (6.1–6.4 kHz at 15°C), and the lower frequency calls of *L. applebyi* Rowley & Cao (4.0–4.3 kHz at 21.5 °C), *L. ardens* Rowley, Tran, Le, Dau, Peloso, Nguyen, Hoang, Nguyen & Ziegler (3.1–3.4 kHz at 21.4–24.7 °C), *L. bidoupensis* Rowley, Le, Tran & Hoang (1.9–3.8 kHz at 19–21 °C), *L. botsfordi* Rowley, Dau & Nguyen (2.6–3.2 kHz at 14  °C), *L. croceus* Rowley, Hoang, Le, Dau & Cao (2.6–3.0 kHz at 21.6–25.1 °C), *L. fuliginosus* Matsui (2.1–2.8 kHz at 19.3–19.6 °C), *L. kalonensis* Rowley, Tran, Le, Dau, Peloso, Nguyen, Hoang, Nguyen & Ziegler (2.8 kHz at 26.4 °C), *L. maculosus* Rowley, Tran, Le, Dau, Peloso, Nguyen, Hoang, Nguyen & Ziegler (2.7–2.8 kHz at 23.3–24.1 °C), *L. melicus* Rowley, Stuart, Neang & Emmett (2.6–4.0 kHz at 26.1–26.2 °C), *L. pallidus* Rowley, Tran, Le, Dau, Peloso, Nguyen, Hoang, Nguyen & Ziegler (2.4–2.7 kHz at 14.0–21.4 °C), *L. pyrrhops* Poyarkov, Rowley, Gogoleva, Vassilieva, Galoyan & Orlov (1.91–2.2 kHz at 25 °C), *L. tadungensis* Rowley, Tran, Le, Dau, Peloso, Nguyen, Hoang, Nguyen & Ziegler (2.6–3.1 kHz at 12.9–22.3 °C) and *L. tuberosus* (2.6–2.8 kHz at 22.5–24.5 °C).

**Table:** 

**Figure 7:**
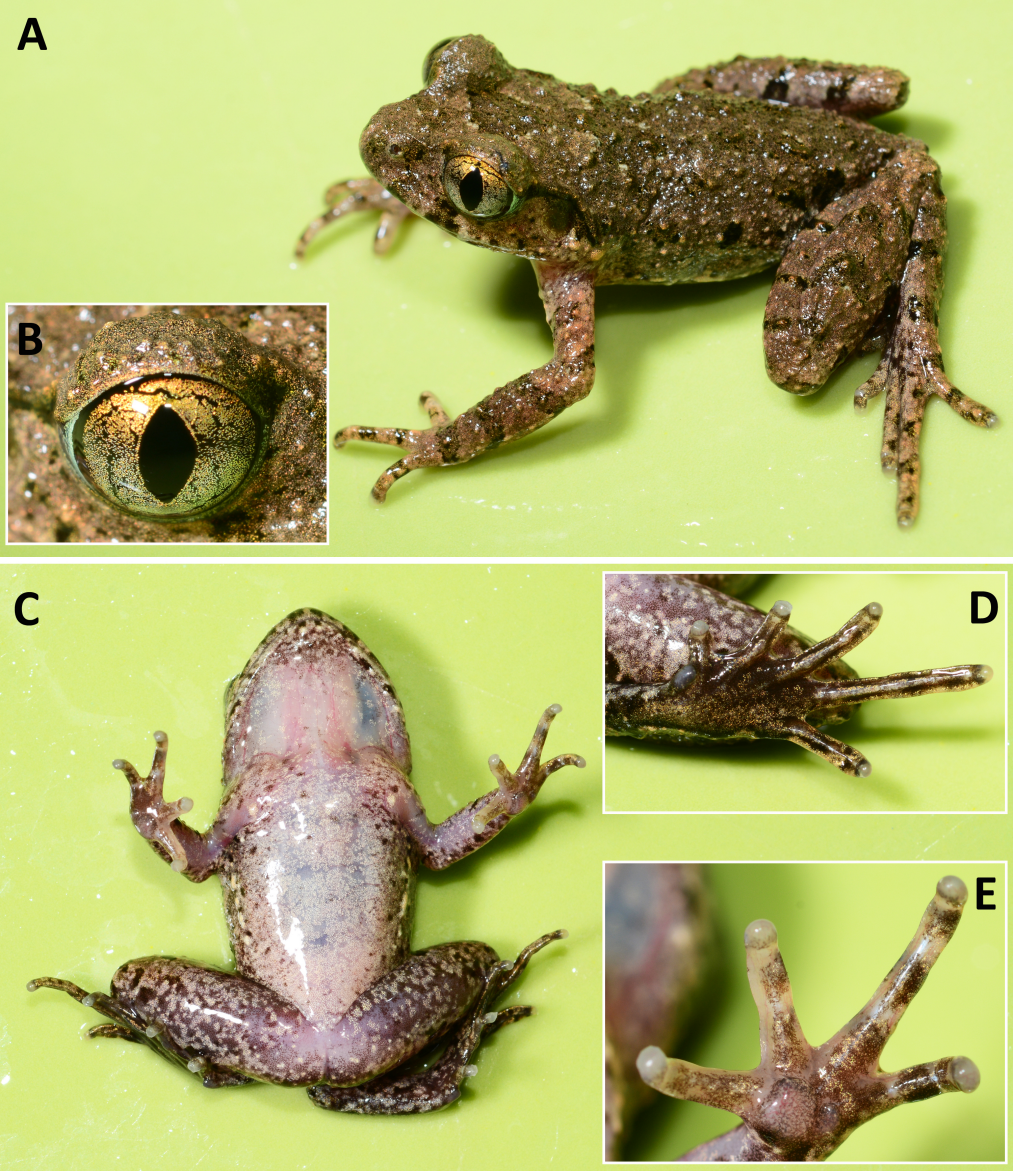
Holotype of *Leptolalax yingjiangensis* sp. nov. (SYS a006532) in life. Holotype of* Leptolalax yingjiangensis* **sp. nov.** (SYS a006532) in life: (A) dorsolateral view; (B) iris coloration; (C) ventral view; (D) plantar view of the left foot; (D) volar view of the left hand. Photos by JH Yang.

**Figure 8:**
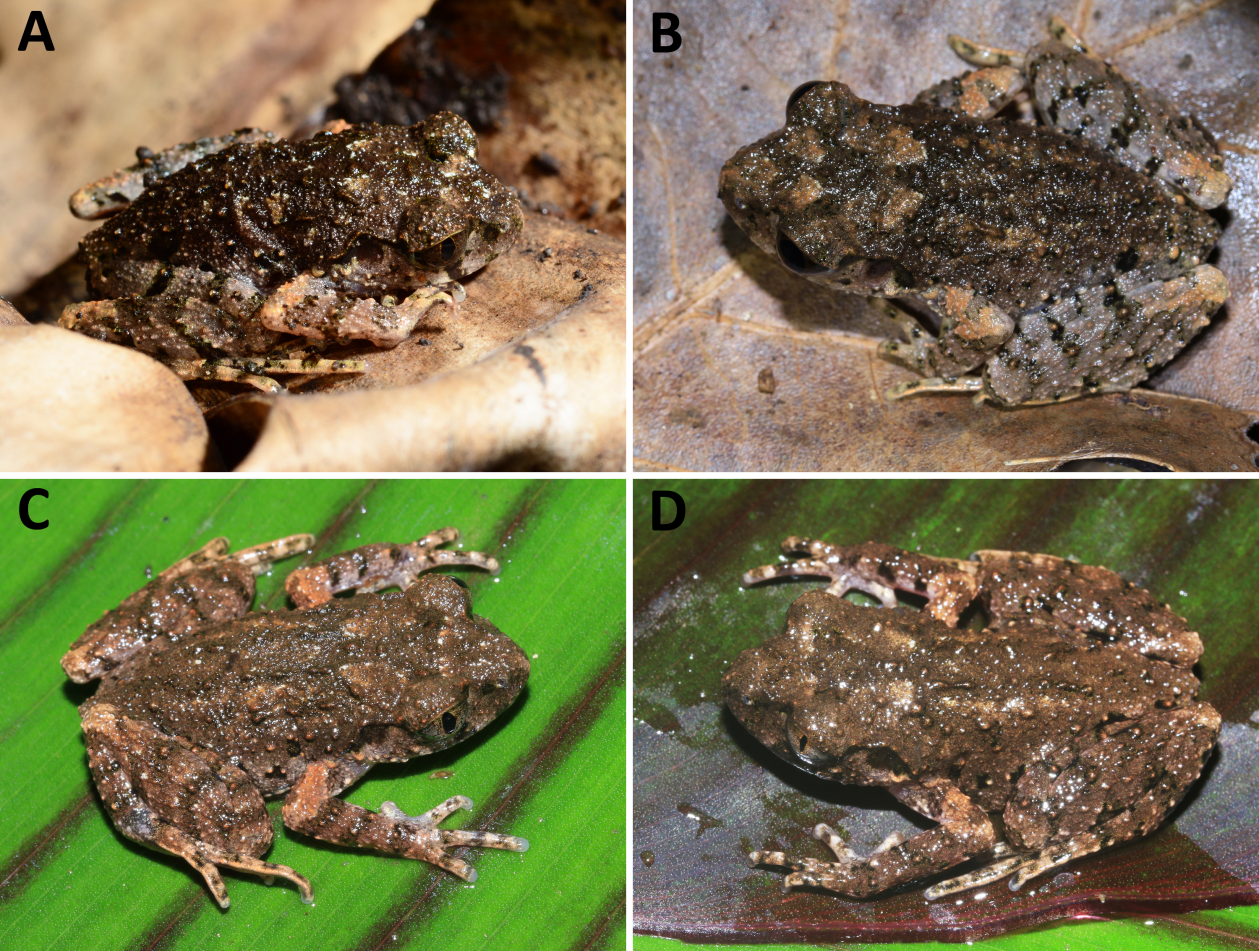
Paratypes of *Leptolalax yingjiangensis***sp. nov.** in life: (A) paratype SYS a006533: (B) paratype SYS a006534; (C) paratype SYS a006536; (D) paratype SYS a006537. Photos by JH Yang.

***Leptolalax yingjiangensis* sp. nov.**
urn:lsid:zoobank.org:act:6D9B19A7-1421-4926-892B-4A3EB666BBC7
Figs. 3, 4, 7 and 8; Tables 2–4

**Holotype.** SYS a006532, adult male, collected from Jinzhuzhai Village, Tongbiguan Town, Yingjiang County, Yunnan Province, China (24°37′33.32″N, 97°37′11.91″E, 1,615 m a.s.l.), on 5 May 2016 by JH Yang.

**Paratypes.** SYS a006533, adult male, data identical to holotype; SYS a006534–a006537, adult males, same locality as holotype, collected on 10 June 2017 by JH Yang.

**Etymology.** The specific name “*yingjiangensis*”, is in reference to the type locality of the new species, Yingjiang County of Yunnan Province, China. For the common name, we suggest “Yingjiang Leaf Litter Toad” (English) and “盈江掌突蟾” (Chinese).

**Diagnosis.** *Leptolalax yingjiangensis* **sp. nov.** can be distinguished from its congeners by a combination of the following characters: (1) small size (SVL 25.7–27.6 mm in males); (2) dorsal skin shagreened and scattered with fine, round brown tubercles; (3) tympanum distinctly discernible, upper half black; (4) fingers webbing absent, and narrow to moderate dermal fringes present on 2nd to 4th fingers; (5) toes with rudimentary webbing and wide lateral fringes; (6) pectoral gland smaller than femoral gland; (7) ventrolateral glands distinct; (8) distinct tiny white flecks present on edges of dark brown markings/blotches on dorsum; (9) flanks with distinct irregular black spots; (10) ventral surface of body creamy white and scattered with distinct small dark brown flecks on chest and lateral sides of belly; (11) iris bicolored, upper half orange yellow, lower half sliver white; (12) a call consisting of a single note and a dominant frequency of 5.7–5.9 kHz (at 19 °C).

**Description of holotype.** SYS a006532, adult male, body size small (SVL 25.7 mm), head width (9.7 mm) slightly shorter than head length (10.3 mm); snout slightly protruding, projecting slightly beyond margin of the lower jaw; nostril equidistant between snout and eye; canthus rostralis gently rounded; loreal region slightly concave; interorbital space flat, larger (IOD 3.0 mm) than upper eyelid (2.8 mm in width) and internarial distance (2.6 mm); pineal ocellus absent; pupil vertical; eye diameter (EYE 3.9 mm) slightly smaller than snout length (SNT 4.2 mm); tympanum distinct, round, diameter (TMP 1.8 mm) smaller than that of the eye, and larger than tympanum-eye distance (TEY 1.1 mm); tympanic rim distinctly elevated relative to skin of temporal region; vomerine teeth absent; vocal sac openings slit-like, located posterolaterally on floor of mouth in close proximity to the margins of the mandible; tongue long, wide, with a small shallow notch at posterior tip; supratympanic ridge distinct, extending from eye to supra-axillary gland; a few indistinct tubercles present on supratympanic ridge. Tips of fingers rounded, slightly swollen; relative finger lengths I = II = IV <  III; nuptial pad absent; subarticular tubercles absent; a large, round inner palmar tubercle distinctly separated from small, round outer palmar tubercle; narrow to moderate dermal fringes present on 2nd to 4th fingers; finger webbing absent. Tips of toes similar to that of fingers; relative toe length I <II <V <III <IV; subarticular tubercles absent; distinct dermal ridges present under the 3rd to 5th toes; large, oval inner metatarsal tubercle present, outer metatarsal tubercle absent; toe webbing rudimentary; wide lateral fringes present on all toes. Tibia 49% of snout-vent length; tibiotarsal articulation reaches to anterior corner of the eye. Skin on dorsum shagreened and scattered with fine, round tubercles; ventral skin smooth; pectoral gland tiny, round, 0.5 mm in diameter, slight smaller than tips of fingers; femoral gland small, round, 1.0 mm in diameter, distinctly larger than pectoral gland and tips of toes, situated on posteroventral surface of thigh, closer to knee than to vent; supra-axillary gland raised, 0.8 in mm diameter; ventrolateral gland distinct as small white dots forming an incomplete line.

**Coloration of holotype in life.** Dorsal surface brown with indistinct dark brown markings, and two indistinct light brown spots on shoulder region (Fig. 7): V-shaped interorbital marking disconnected to the W-shaped marking between axillae; distinct tiny white flecks present on edges of dark brown markings/blotches on dorsal surfaces of head and body; fine, brown tubercles present on upper eyelids, snout, head, dorsal surfaces of body and limbs; upper lip with black large blotches and small spots; lower margin of supratympanic ridge black; upper half of tympanum dark brown; a distinct black spot present on loreal region; narrow transverse dark brown cross-bars present on dorsal surface of limbs; a few distinct irregular black spots present on flanks from groin to axilla, the one on groin largest; elbow and upper arms in light orange brown coloration; fingers and toes with transverse bars. Venter creamy white and scattered with dark brown flecks on chest and lateral sides of belly; medial belly immaculate; throat transparent pinkish; margin of lower lip scattered with dark brown spots and flecks; ventral surfaces of thigh pinkish and mottled with white and dark brown. Supra-axillary gland light brown; pectoral white and indistinct; femoral and ventrolateral glands white and distinct. Iris bicolored, upper half orange yellow, lower half sliver white.

**Coloration of holotype in preservative.** Dorsum turned dark brown with slightly paler limbs (Fig. 4). Dark brown markings on dorsum indistinct; black spots on flanks and transverse cross-bars on limbs still distinct; white flecks on dorsum still visible; ventral surface creamy white and scattered with dark brown flecks on chest and lateral sides of belly; throat and medial belly almost immaculate; thigh mottled with creamy white and dark brown; pectoral gland nearly invisible; femoral and ventrolateral glands still visible.

**Variations.** All five paratypes (Figs. 4 & 8) match the overall characters of the holotype (for measurements of the type series see Table 2). Most types have narrow to moderate dermal fringes on 2nd to 4th fingers, except that narrow fringes only present on 2nd and 3rd fingers in paratypes SYS a006533 and SYS a00654. Tibiotarsal articulation reaches to loreal region in two paratypes SYS a006534 and SYS a006535.

**Distribution and natural history.** *Leptolalax yingjiangensis* **sp. nov.** is currently only known from its type locality in Yingjiang County of Yunnan Province, China. The male holotype was found calling and perching under leaf litter nearby a small clear-water rocky stream (exactly the same stream as types of *Leptolalax purpurus*), on 5 May 2016, and very few male calls were detected during the survey. While during the survey on 10 June 2017, calling males of the new species were ubiquitous along the stream and riparian forest, and four calling males were collected. No male calls and individuals of the new species were detected during other two night surveys on 8 December 2016 and 20 April 2017.

**Advertisement calls.** The call of an unvouchered individual of *L. yingjiangensis* was recorded at an ambient temperature of 19 °C on 10 June 2017. The call series was composed of a series of uniform and continuous single-note calls (Fig. 5C). Calls were repeated in series at a mean rate of 5.8 times per second. Each call had a duration of 0.028–0.042 s (mean 0.036 ± 0.003 s, *N* = 35) with a peak frequency of 5.7–5.9 kHz. The intercall interval was relatively stable and had a duration of 0.113–0.174 s (mean 0.134 ± 0.017 s, *N* = 35) (Table 3).

**Comparison.** By the presence of supra-axillary and ventrolateral glands, *Leptolalax yingjiangensis* **sp. nov.** can be allocated into the subgenus *Lalos*, and distinctly differs from the 15 known species of the subgenus *Leptolalax*, i.e., *L. arayai*, *L. dringi*, *L. fritinniens*, *L. gracilis*, *L. hamidi*, *L. heteropus*, *L. kajangensis*, *L. kecil*, *L. marmoratus*, *L. maurus*, *L. melanoleucus*, *L. pictus*, *L. platycephalus*, *L. sabahmontanus* and *L. solus*, all of which occur south of the Isthmus of Kra and lack supra-axillary and ventrolateral glands (Dubois et al., 2010; Dehling & Matsui, 2013; Matsui, Zainudin & Nishikawa, 2014). *Leptolalax yingjiangensis* **sp. nov.** differs from all other species in the subgenus *Lalos* by having brown dorsum in life, pectoral gland indistinct and smaller than femoral gland, narrow to moderate dermal fringes present on 2nd to 4th fingers; distinct small white flecks present on dorsum, iris bicolored, as well as a combination of male body size, presence of black spots on the flank, plus ventral coloration, degree of webbing and fringing on the toes, and dorsal skin texture (See Table 4 for a summarized comparison with all species in the subgenus *Lalos*).

*Leptolalax yingjiangensis* **sp. nov.** differs from the phylogenetically close congener, *L. khasiorum* Das, Tron, Rangad & Hooroo, by having head slightly longer than wide (vs. head wider than long in *khasiorum*), a comparatively small tympanum (males TMP/EYE ratio 0.41–0.46 vs. 0.47–0.55 in *khasiorum*), supra-axillary gland and small tubercles on dorsum and hind limbs brown in life (vs. pinkish-red in *khasiorum*), upper parts of iris orange yellow in life (vs. bright orange in *khasiorum*), and pectoral gland distinctly smaller than femoral gland (vs. reversed condition in *khasiorum*).

*Leptolalax yingjiangensis* **sp. nov.** differs from the phylogenetically close congener, *L. puhoatensis*, by having ventral surface creamy white with dark brown flecks on chest and margins in males (vs. reddish brown with white dusting in *puhoatensis*), absence of skin folds on dorsum (vs. low skin folds present on dorsum in life in *puhoatensis*), wide dermal fringes on toes (vs. narrow in *puhoatensis*), dorsal coloration brown in life (vs. dark reddish brown in *puhoatensis*).

*Leptolalax yingjiangensis* **sp. nov.** differs from the phylogenetically close congener, *L. petrops*, by having dermal fringes present on fingers (vs. dermal fringes absent on fingers in *puhoatensis*), toes webbing rudimentary (vs. absent in *petrops*), and wide dermal fringes on toes (vs. narrow in *puhoatensis*), ventral surface creamy white with dark brown flecks on chest and lateral sides of belly (vs. immaculate white in *petrops*), and dorsal skin shagreened scattered with small tubercles (vs. dorsal skin highly tuberculate in *petrops*).

*Leptolalax yingjiangensis* **sp. nov.** further differs from the sympatric *L. ventripunctatus* by having dermal fringes present on fingers (vs. dermal fringes absent on fingers in *ventripunctatus*), wide dermal fringes on toes (vs. absent or narrow in *ventripunctatus*), absence of longitudinal skin folds on dorsum of body (vs. present in *ventripunctatus*), pectoral gland smaller than tips of fingers and femoral grand (vs. reversed condition in *ventripunctatus*), distinct white tiny flecks present on dorsum (vs. such white flecks absent in *ventripunctatus*), medial belly immaculate creamy white (vs. distinct small black spots present on belly in *ventripunctatus*, see Fig. 3).

*Leptolalax yingjiangensis* **sp. nov.** further differs from the sympatric *L. purpurus* by having dermal fringes present on fingers (vs. dermal fringes absent on fingers in *purpurus*), tibiotarsal articulation reaches to anterior corner of the eye or loreal region (vs. reaches posterior corner of the eye in *purpurus*), a relatively longer hindlimb (males TIB/SVL ratio 0.47–0.49 in *yingjiangensis* **sp. nov.** vs. 0.43–0.45 in *purpurus*; males HLL/SVL ratio 1.57–1.65 in *yingjiangensis* **sp. nov.** vs. 1.41–1.48 in *purpurus*), pectoral gland smaller than tips of fingers and femoral grand (vs. reversed condition in *purpurus*), dorsal surface brown in life (vs. purplish brown in *purpurus*, see Fig. 3), distinct white tiny flecks present on dorsum (vs. absent in *purpurus*), ventral surface creamy white and scattered with small black spots on chest and lateral sides of belly (vs. dull white with grey dusting in *purpurus*, see Figs. 3 and 4).

From the rest two known *Leptolalax* species from Yunnan, *Leptolalax yingjiangensis* **sp. nov.** differs from *L. alpinus* by having dermal fringes present on fingers (vs. dermal fringes absent on fingers in *alpinus*), pectoral gland smaller than tips of fingers and femoral grand (vs. reversed condition in *alpinus*), and medial belly immaculate creamy white (vs. distinct dark brown spots/blotches present on belly in *alpinus*, see Figs. 6C–6D); *Leptolalax yingjiangensis* **sp. nov.** further differs from *L. tengchongensis* by dermal fringes present on fingers (vs. dermal fringes absent on fingers in *tengchongensis*), wide dermal fringes on toes (vs. narrow in *tengchongensis*), small tubercles on dorsum in brown coloration in life (vs. reddish in *tengchongensis*), distinct white tiny flecks present on dorsum (vs. such white flecks absent in *tengchongensis*), medial belly immaculate creamy white (vs. distinct dark brown blotches present on chest and belly in *tengchongensis*, see Figs. 6E–6F), and a bicolored iris (vs. uniform coloration in *tengchongensis*).

*Leptolalax yingjiangensis* **sp. nov.** further differs from *L. pelodytoides*, the only known species from adjoining northeast Myanmar, by having dermal fringes present on fingers (vs. dermal fringes absent on fingers in *pelodytoides*), rudimentary toes webbing (vs. wide in *pelodytoides*), wide dermal fringes on toes (vs. narrow in *pelodytoides*), dermal ridges under toes distinct (vs. poorly distinct in *pelodytoides*), ventral surface creamy white with dark brown flecks on chest and margins (vs. immaculate whitish in *pelodytoides*), and the absence of longitudinal skin folds on dorsum (vs. present in *pelodytoides*).

In addition to morphological differences, the advertisement call of *Leptolalax yingjiangensis* **sp. nov.** further differs from all other congeners in the subgenus *Lalos* for which comparable acoustic data are available in having a call consisting invariably of a single note. Of the *Leptolalax* species in the region with known calls, only *L. purpurus*, *L. tuberosus* and *L. puhoatensis* are reported having an invariably single-note call, but the call duration (28–42 ms at 19 °C) of *Leptolalax yingjiangensis* **sp. nov.** still can be separated from that of *L. purpurus* (86–117 ms at 15 °C), *L. tuberosus* (54–78 ms at 22.4–22.5 °C), and *L. puhoatensis* (6–14 ms at 22.3–25.8 °C). In addition, the dominant frequency of 5.7–5.9 kHz (at 19 °C) further distinguishes the call of *Leptolalax yingjiangensis* **sp. nov.** from that of the higher frequency calls of *L. aereus* (6.2–7.9 kHz at 22.4–25.7 °C), *L. isos* (5.9–6.2 kHz at 22.4–22.8 °C), and *L. ventripunctatus* (6.1–6.4 kHz at 15 °C), and the lower frequency calls of *L. applebyi* (4.0–4.3 kHz at 21.5 °C), *L. ardens* (3.1–3.4 kHz at 21.4–24.7 °C), *L. bidoupensis* (1.9–3.8 kHz at 19–21 °C), *L. botsfordi* (2.6–3.2 kHz at 14 °C), *L. croceus* (2.6–3.0 kHz at 21.6–25.1 °C), *L. fuliginosus* (2.1–2.8 kHz at 19.3–19.6 °C), *L. kalonensis* (2.8 kHz at 26.4 °C), *L. maculosus* (2.7–2.8 kHz at 23.3–24.1 °C), *L. melicus* (2.6–4.0 kHz at 26.1–26.2 °C), *L. pallidus* (2.4–2.7 kHz at 14.0–21.4 °C), *L. puhoatensis* (4.9–5.6 kHz at 22.3–25.8°C), *L. purpurus* (4.3–4.5 kHz at 15 °C), *L. pyrrhops* (1.91–2.2 kHz at 25 °C), *L. tadungensis* (2.6–3.1 kHz at 12.9–22.3 °C) and *L. tuberosus* (2.6–2.8 kHz at 22.5–24.5 °C).

**Figure 9:**
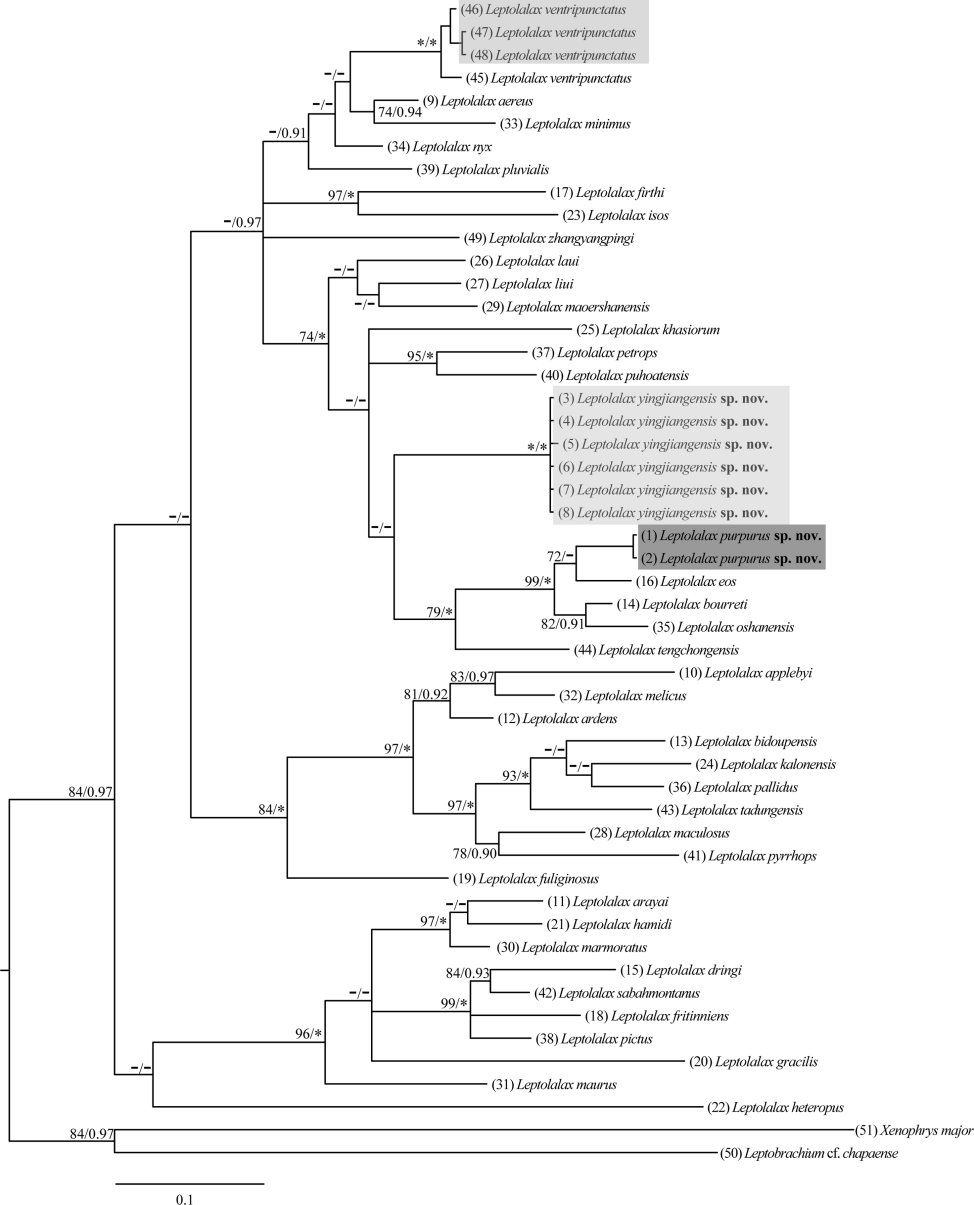
Molecular analysis. Maximum-likelihood phylogram of the genus * Leptolalax* from partial DNA sequences of the mitochondrial 16S rRNA gene. Number at the node is bootstrap support/posterior probability (BS/PP). “*” means BS is 100 or PP is 1.00, “-” means BS less than 70 or PP less than 0.90.

**Table 5:** Genetic distances data. Uncorrected *p*-distances among *Leptolalax* species and outgroups based on 16S rRNA fragment.

		1–2	3–8	9	10	11	12	13	14	15	16	17	18	19	20	21	22	23	24	25	26	27	28	29	30	31
1–2	*L. purpurus* **sp.nov.**	0.0																								
3–8	*L. yingjiangensis* **sp. nov.**	7.8	0.0																							
9	*L. aereus*	9.0	10.7	–																						
10	*L. applebyi*	12.0	13.5	13.1	–																					
11	*L. arayai*	14.4	13.4	14.5	12.3	–																				
12	*L. ardens*	13.1	12.8	12.1	6.5	11.4	–																			
13	*L. bidoupensis*	13.4	13.1	14.2	8.4	15.1	8.1	–																		
14	*L. bourreti*	3.8	8.1	9.1	12.3	14.0	12.7	15.1	–																	
15	*L. dringi*	15.8	16.9	14.7	14.6	10.0	13.7	15.8	14.7	–																
16	*L. eos*	3.2	7.7	10.0	11.7	13.6	13.0	13.7	3.5	16.4	–															
17	*L. firthi*	11.6	14.0	10.7	15.4	16.5	15.1	17.0	11.0	16.5	11.9	–														
18	*L. fritinniens*	15.3	17.5	15.4	16.4	10.3	14.0	14.4	16.4	6.9	16.4	17.5	–													
19	*L. fuliginosus*	12.4	12.4	12.1	9.7	15.2	9.7	10.0	11.7	14.4	11.3	16.9	14.4	–												
20	*L. gracilus*	20.7	19.2	16.6	16.1	9.7	14.8	17.2	18.3	11.3	19.8	19.8	12.0	18.1	–											
21	*L. hamidi*	14.0	13.7	13.7	13.0	3.8	11.7	16.2	13.6	9.7	13.3	16.5	7.7	14.9	10.0	–										
22	*L. heteropus*	19.8	19.4	16.9	15.4	18.3	16.2	16.1	19.8	18.5	19.8	20.9	17.8	16.8	20.4	17.5	–									
23	*L. isos*	10.6	11.6	11.0	13.3	14.7	12.7	13.3	9.3	16.1	10.6	10.8	17.1	12.6	19.0	14.7	19.0	–								
24	*L. kalonensis*	15.8	14.1	15.5	11.7	15.4	9.7	5.9	16.9	14.7	16.5	19.5	14.7	12.3	18.7	16.5	20.4	16.1	–							
25	*L. khasiorum*	10.7	8.8	12.7	13.3	15.8	14.4	14.7	10.4	16.4	10.4	11.6	19.3	13.8	19.9	16.2	17.9	14.0	17.2	–						
26	*L. laui*	7.8	7.4	8.8	14.5	14.7	13.1	14.4	8.1	15.4	7.2	11.3	17.2	13.1	18.8	15.5	19.1	11.4	16.6	8.7	–					
27	*L. liui*	7.4	8.4	8.1	13.8	15.1	14.2	13.0	7.8	15.4	6.8	11.0	16.4	12.4	20.8	15.8	18.7	11.4	14.4	9.1	5.3	–				
28	*L. maculosus*	13.3	13.0	11.7	9.4	15.1	5.9	6.9	13.0	14.7	13.7	14.7	16.5	11.3	18.3	16.2	16.8	13.3	8.4	14.3	13.0	12.3	–			
29	*L. maoershanensis*	8.8	8.8	6.8	12.7	13.3	12.4	12.3	8.5	16.1	8.4	13.7	16.8	12.1	19.1	14.4	17.9	11.7	14.4	10.8	5.9	5.3	12.3	–		
30	*L. marmoratus*	13.6	13.7	14.0	10.9	2.9	11.3	15.1	13.3	8.7	12.9	14.6	8.7	14.8	10.0	3.8	17.1	15.0	15.4	14.0	15.1	14.0	14.7	13.6	–	
31	*L. maurus*	14.7	15.8	14.4	12.4	8.7	12.3	16.1	14.7	9.6	15.1	17.1	10.9	15.9	11.6	9.0	18.3	14.7	17.5	15.8	15.4	15.4	15.8	14.7	8.7	–
32	*L. melicus*	12.4	13.5	11.1	5.6	11.7	4.4	7.2	12.7	13.3	12.7	15.1	14.3	9.7	14.0	11.3	15.1	13.7	10.0	14.4	14.2	14.5	8.1	13.4	11.7	12.7
33	*L. minimus*	9.7	9.4	5.0	13.4	15.5	13.4	14.4	8.4	16.1	9.0	10.6	16.8	12.4	18.8	16.2	18.4	12.4	16.9	11.0	7.8	8.4	13.0	7.5	14.7	15.5
34	*L. nyx*	8.1	10.0	3.8	12.0	14.0	11.4	12.7	8.1	15.0	8.7	8.7	15.4	11.4	18.1	14.0	16.2	10.3	14.8	10.6	7.5	6.8	11.0	7.5	13.0	14.3
35	*L. oshanensis*	4.7	9.0	9.4	13.0	15.8	12.3	15.4	3.5	14.7	4.7	11.0	16.4	11.7	19.1	14.7	20.2	9.6	17.2	10.0	6.2	7.5	12.9	9.1	15.0	14.7
36	*L. pallidus*	14.0	12.7	13.8	9.0	13.0	8.4	5.3	15.4	15.4	13.7	16.6	14.0	11.0	16.5	14.8	18.3	15.5	7.1	15.8	13.7	13.7	8.8	13.3	13.0	15.1
37	*L. petrops*	8.7	7.2	9.3	14.5	14.7	14.1	15.5	9.0	16.0	9.4	11.3	18.2	12.7	19.0	15.8	19.4	11.6	16.2	9.0	9.1	8.7	12.6	9.7	15.4	15.4
38	*L. pictus*	16.8	16.1	15.8	13.6	9.0	14.0	15.4	16.1	5.6	16.0	15.4	5.3	14.4	10.7	8.7	18.2	17.5	14.7	16.8	16.8	16.1	15.4	16.1	7.4	10.3
39	*L. pluvialis*	9.7	10.0	5.3	12.3	15.8	13.1	13.4	9.4	16.0	10.0	11.3	16.4	12.4	18.3	15.8	15.7	12.3	15.2	11.0	7.8	6.5	12.3	5.9	14.0	15.7
40	*L. puhoatensis*	8.7	9.4	10.0	14.1	14.0	12.6	14.3	7.4	15.3	9.4	10.6	18.2	13.0	19.0	15.4	18.2	10.6	15.4	9.7	7.4	8.7	11.9	8.4	13.9	14.3
41	*L. pyrrhops*	15.8	14.0	13.0	10.7	15.4	8.1	8.4	16.1	16.5	14.7	16.5	15.7	11.9	16.4	15.8	15.7	13.7	10.9	15.8	14.1	13.7	7.8	13.7	14.6	17.2
42	*L. sabahmontanus*	16.5	16.8	15.4	12.2	10.0	12.6	14.7	15.1	5.3	16.4	16.5	6.8	12.6	12.0	9.0	20.8	17.2	13.3	18.3	17.3	16.5	14.0	16.5	8.4	9.6
43	*L. tadungensis*	13.4	14.5	13.1	8.7	16.2	7.4	5.0	15.2	15.1	14.8	15.5	13.3	10.3	18.4	17.3	16.4	13.0	6.5	14.7	14.1	12.7	7.1	13.0	15.1	15.8
44	*L. tengchongensis*	7.5	7.8	8.4	14.2	14.0	13.1	14.8	7.1	15.7	6.8	10.6	16.4	13.5	19.5	13.7	19.4	9.6	16.6	9.7	6.5	8.7	12.0	8.7	14.0	15.1
45	*L. ventripunctatus*	10.3	12.3	7.1	15.4	15.0	13.7	16.2	11.0	16.4	11.3	10.7	16.4	14.1	21.0	15.8	17.9	11.0	18.4	13.0	10.4	9.4	14.0	9.4	14.3	16.1
46–48	*L. ventripunctatus*	9.6	12.3	6.5	15.5	15.1	13.7	16.3	10.3	16.1	10.6	10.0	16.1	14.1	20.7	15.8	17.9	11.0	18.4	12.3	9.7	8.7	13.3	9.4	14.0	15.7
49	*L. zhangyangpingi*	10.4	12.1	10.8	14.5	18.4	15.6	14.5	11.4	19.0	10.7	13.0	19.5	13.5	21.5	18.4	20.2	12.3	17.1	13.1	9.8	10.1	13.7	11.8	16.9	17.2
50	*Leptobrachium* cf. *chapaense*	24.6	22.2	22.5	22.0	20.9	22.3	25.4	24.5	24.7	24.2	25.1	25.5	23.8	25.2	22.5	24.9	22.4	26.9	21.4	22.5	22.9	22.8	21.7	20.9	21.0
51	*Xenophrys major*	26.9	23.7	25.8	24.2	22.7	22.1	25.3	25.6	25.4	25.3	26.4	27.1	27.9	24.4	24.8	25.3	23.2	25.8	25.7	25.5	25.1	24.0	26.2	23.5	22.4

		32	33	34	35	36	37	38	39	40	41	42	43	44	45	46–48	49	50	51
1–2	*L. purpurus* **sp.nov.**																		
3–8	*L. yingjiangensis* **sp. nov.**																		
9	*L. aereus*																		
10	*L. applebyi*																		
11	*L. arayai*																		
12	*L. ardens*																		
13	*L. bidoupensis*																		
14	*L. bourreti*																		
15	*L. dringi*																		
16	*L. eos*																		
17	*L. firthi*																		
18	*L. fritinniens*																		
19	*L. fuliginosus*																		
20	*L. gracilus*																		
21	*L. hamidi*																		
22	*L. heteropus*																		
23	*L. isos*																		
24	*L. kalonensis*																		
25	*L. khasiorum*																		
26	*L. laui*																		
27	*L. liui*																		
28	*L. maculosus*																		
29	*L. maoershanensis*																		
30	*L. marmoratus*																		
31	*L. maurus*																		
32	*L. melicus*	–																	
33	*L. minimus*	12.4	–																
34	*L. nyx*	10.1	4.4	–															
35	*L. oshanensis*	13.0	7.7	7.7	–														
36	*L. pallidus*	9.1	13.0	13.0	14.7	–													
37	*L. petrops*	14.9	10.0	8.7	9.7	14.7	–												
38	*L. pictus*	13.7	15.7	14.7	16.8	14.4	16.0	–											
39	*L. pluvialis*	12.4	6.5	5.3	10.0	13.7	9.7	15.7	–										
40	*L. puhoatensis*	12.7	8.1	7.7	8.1	15.0	6.2	16.4	8.7	–									
41	*L. pyrrhops*	11.0	14.7	13.3	15.4	8.1	15.0	15.8	14.4	16.4	–								
42	*L. sabahmontanus*	10.9	15.7	15.0	15.8	13.0	17.5	4.4	16.8	15.7	15.0	–							
43	*L. tadungensis*	9.1	13.4	12.3	14.8	6.5	14.4	15.1	12.0	14.0	8.0	14.0	–						
44	*L. tengchongensis*	13.5	7.1	7.4	6.8	14.4	6.5	15.7	9.7	7.1	15.8	17.2	14.5	–					
45	*L. ventripunctatus*	13.7	7.7	5.6	10.3	16.6	11.3	17.2	7.4	9.9	14.7	17.2	14.8	10.6	–				
46–48	*L. ventripunctatus*	13.8	7.1	5.0	9.7	16.6	10.6	16.8	6.8	9.3	14.7	16.8	14.4	10.0	0.6	–			
49	*L. zhangyangpingi*	15.6	11.0	9.1	11.0	16.6	12.0	17.9	9.4	11.3	15.9	18.7	15.6	9.7	11.0	10.4	–		
50	*Leptobrachium* cf. *chapaense*	24.3	22.5	22.0	23.7	23.3	20.5	24.3	22.1	21.6	20.9	25.5	24.1	23.8	20.8	20.8	27.6	–	
51	*Xenophrys major*	23.7	27.8	25.3	26.1	26.9	24.1	25.0	24.5	25.3	26.5	26.2	25.8	26.1	25.7	24.9	28.3	26.8	–

**Molecular relationships.** For the 535 base pairs of the 16S rRNA from 51 individuals, both ML and BI phylogenetic analyses fully supported our hypothesis that the *Leptolalax* species collected from Yingjiang represent three different clades with relatively high bootstrap support (BS) and strong posterior probability (PP) (Fig. 9). Our newly collected samples of *L. ventripunctatus* from Yingjiang had clustered in the same clade with *L. ventripunctatus* sensu stricto with strong support (100/1.00 for BS/PP). All six specimens of *Leptolalax purpurus* **sp. nov.** were clustered together into a highly divergent lineage (100/1.00 for BS/PP); however the phylogenetic position of this species was not well resolved in both ML and BI analyses since only a small fragment of mtDNA was used in the analysis. *Leptolalax yingjiangensis* **sp. nov.** was clustered into the clade comprising *L. bourreti, L. eos* and *L. oshanensis* (99/1.00 in BS/PP analyses) .

For the uncorrected *p*-distances among and within the 16S rRNA mtDNA gene fragments of the studied *Leptolalax* taxa (Table 5), the observed interspecific distances range from *p* = 2.9% (between *L. arayai* and *L. marmoratus*) to 21.5% (between *L. gracilis* and *L. zhangyangpingi* Jiang, Yan, Suwannapoom, Chomdej & Che). Our samples of *L. ventripunctatus* were genetically close to *L. ventripunctatus* s.s. with a low *p*-distance (*p* = 0.6%); and these specimens also match the diagnostic characters of *L. ventripunctatus* s.s. so that we allocated them to *L. ventripunctatus*. *Leptolalax yingjiangensis* **sp. nov.** differed from all other congeners by remarkably high genetic distances between 7.2% to 19.4%, with the lowest value *p* = 7.2% observed in the comparison with the sequence of *L. petrops* which was still significantly higher than those observed between several pairs of well-distinguished species of *Leptolalax*. *Leptolalax purpurus* **sp. nov.** differed from all other congeners with genetic distances between 3.2% to 20.7%, with the lowest value *p* = 3.2% observed in the comparison with the sequence of *L. eos*. This value was slightly higher than the lowest interspecific *p*-distance (*p* = 2.9%, between *L. arayai* and *L. marmoratus*) among species of *Leptolalax* examined and the value (3% value of *p*-distance in 16S rRNA) that usually represents differentiation at the species levels of anurans (Vences et al., 2005; Fouquet et al., 2007).

## Discussion

In the genus *Leptolalax*, pairs of sympatric species are commonly reported among tropical members in Indochina, e.g., *L. eos* and *L. ventripunctatus*, *L. pluvialis* Ohler, Marquis, Swan & Grosjean and *L. bourreti*, *L. applebyi* and *L. tuberosus*, *L. croceus* and *L. applebyi*, *L. eos* and *L. puhoatensis* (Rowley & Cao, 2009; Rowley et al., 2010a; Ohler et al., 2011; Rowley, Dau & Cao, 2017). Our results revealed a rare case that three species of *Leptolalax* co-occur in the same stream, which also represents the first record of sympatric *Leptolalax* species from China. According to our limited preliminary data (Table 6), the breeding season of *L. purpurus* starts rather early (probably in February) and ends in April; *L. yingjiangensis* breeds relatively later starting from May, with the peak in June; *L. ventripunctatus* presents the longest breeding period among the three species, with the peak in April. Thus, we assumed that the main breeding seasons of the three sympatric *Leptolalax* in Yingjiang County should be different but have overlapping, which needs to be verified by future study. Our survey showed that these three sympatric species overlap in time and space, but we have no data on the resources they utilize, such as food composition, trophic structure and other parameters. Future ecological studies are needed in order to understand the interspecific competition and mechanisms of niche segregation among the sympatric species of *Leptolalax*.

**Table 6:** Activity pattern of the three sympatric *Leptolalax* species. Male calls detected and number of individuals in the study site during four field surveys in between 2016 and 2017.

	8 Dec 2016	20–21 Apr 2017	5 May 2016	10–11 Jun 2017
*L. ventripunctatus*	–/0	+ + + ∕10	+∕4	+∕2
*L. yingjiangensis*	–/0	–/0	+ + ∕2	+ + + ∕8
*L. purpurus*	–/1	+∕1	–/0	–/0

**Notes.**

Remarks: Characters above branches indicate male calls detected: − − represents no calling males were detected; + represents 1–5 calling males detected; + + represents 6–10 calling males detected; + +  + represents more than 10 calling males detected. Numbers below branches indicate individuals found.

The type locality of *L. purpurus* and *L. yingjiangensis* is less than one kilometer from the boundary of Tongbiguan Nature Reserve, and ca. 5 km from the international border with Myanmar’s Kachin State (Fig. 1); and we can expect that these two new species and *L. ventripunctatus* also occur in the adjacent well-preserved natural forests of Tongbiguan Nature Reserve and Kachin State. The discovery of *L. purpurus* and *L. yingjiangensis* brings the total number of *Leptolalax* recorded from China up to eleven, which further reveals that the currently known species diversity of the genus *Leptolalax* from China and the region is still highly underestimated (Sung, Yang & Wang, 2014; Rowley et al., 2015b; Yang et al., 2016), and warrants further attention from field herpetologists and taxonomists.

While most species of *Leptolalax* have small and restricted distribution areas (Ohler et al., 2011; Rowley et al., 2016), our field surveys reveal that *L. ventripunctatus* has a relatively wide distribution ranging from northern Vietnam, northern Laos to southern Yunnan of China (Fei et al., 2009; Ohler et al., 2011; Rowley, Dau & Cao, 2017; this study), and it is also found sympatrically with other *Leptolalax* species in other localities: sympatric with *L. eos* in northern Laos (Ohler et al., 2011), and with *L. puhoatensis* and *L. eos* in northern Vietnam (Rowley, Dau & Cao, 2017).

##  Supplemental Information

10.7717/peerj.4586/supp-1 Supplemental Information 1 New DNA sequences in this paper

10.7717/peerj.4586/supp-2 Appendix S1 Checklist of currently recognized 53 species of *Leptolalax* and the literature referred for comparative morphological data of each species

10.7717/peerj.4586/supp-3 Appendix S2 Specimens of *Leptolalax* examined in this study

## Additional Information and Declarations

### DNA Deposition

The following information was supplied regarding the deposition of DNA sequences: The new sequences analysed in this study are accessible via GenBank accession numbers MG520350–MG520361.

### Data Availability

The following information was supplied regarding data availability: Specimens of the two new species described in this paper are deposited at the Museum of Biology, Sun Yat-sen University, Guangzhou, China.

### New Species Registration

The following information was supplied regarding the registration of a newly described species: Publication LSID urn:lsid:zoobank.org:pub:31C06247-A201-42AD-8FF8- B6510530C39D; *Leptolalax purpurus* sp. nov. urn:lsid:zoobank.org:act:40673093-C3A4-4F0C-AD60-C0B517FCEF70; *Leptolalax yingjiangensis* sp. nov. urn:lsid:zoobank.org:act:6D9B19A7-1421-4926-892B-4A3EB666BBC7.
