# Supplementary material for: Description of two new sympatric species of the genus Leptolalax (Anura: Megophryidae) from western Yunnan of China
Source: PeerJ. 2018 Apr 10;6:e4586. doi: 10.7717/peerj.4586 (PMC5898428; doi:10.7717/peerj.4586)
Supplement: Appendix S1 [file peerj-06-4586-s002.docx]

**Appendix S1:** Checklist of currently recognized 53 species of *Leptolalax* and the literature referred for comparative morphological data of each species.

| Taxon | References |
| --- | --- |
| *Leptolalax aereus* Rowley, Stuart, Richards, Phimmachak, & Sivongxay | Rowley et al., 2010c |
| *Leptolalax alpinus* Fei, Ye, & Li, 1990 | Fei et al., 2009; Fei, Ye & Jiang, 2012 |
| *Leptolalax applebyi* Rowley & Cao, 2009 | Rowley & Cao, 2009 |
| *Leptolalax arayai* Matsui, 1997 | Matsui, 1997 |
| *Leptolalax ardens* Rowley, Tran, Le, Dau, Peloso, Nguyen, Hoang, Nguyen, & Ziegler, 2016 | Rowley et al., 2016 |
| *Leptolalax bidoupensis* Rowley, Le, Tran, & Hoang, 2011 | Ohler et al., 2011 |
| *Leptolalax botsfordi* Rowley, Dau, & Nguyen, 2013 | Rowley et al., 2011 |
| *Leptolalax bourreti* Dubois, 1983 | Dubois, 1983; Ohler et al., 2011 |
| *Leptolalax croceus* Rowley, Hoang, Le, Dau, & Cao, 2010 | Rowley et al., 2010a |
| *Leptolalax dringi* Dubois, 1987 | Inger, Stuebing & Tan, 1995; Matsui & Dehling, 2012 |
| *Leptolalax eos* Ohler, Wollenberg, Grosjean, Hendrix, Vences, Ziegler, & Dubois, 2011 | Ohler et al., 2011 |
| *Leptolalax firthi* Rowley, Hoang, Dau, Le, & Cao, 2012 | Rowley et al., 2012 |
| *Leptolalax fritinniens* Dehling & Matsui, 2013 | Dehling & Matsui, 2013 |
| *Leptolalax fuliginosus* Matsui, 2006 | Matsui, 2006 |
| *Leptolalax gracilis* (Günther, 1872) | Günther, 1872; Dehling, 2012b |
| *Leptolalax hamidi* Matsui, 1997 | Matsui, 1997 |
| *Leptolalax heteropus* (Boulenger, 1900) | Boulenger, 1900 |
| *Leptolalax isos* Rowley, Stuart, Neang, Hoang, Dau, Nguyen, & Emmett, 2015 | Rowley et al., 2015a |
| *Leptolalax kajangensis* Grismer, Grismer, & Youmans, 2004 | Grismer, Grismer & Youmans, 2004. |
| *Leptolalax kalonensis* Rowley, Tran, Le, Dau, Peloso, Nguyen, Hoang, Nguyen, & Ziegler, 2016 | Rowley et al., 2016 |
| *Leptolalax kecil* Matsui, Belabut, Ahmad, & Yong, 2009 | Matsui et al., 2009 |
| *Leptolalax khasiorum* Das, Tron, Rangad, & Hooroo, 2010 | Das et al., 2010 |
| *Leptolalax lateralis* (Anderson, 1871) | Anderson, 1871; Humtsoe et al., 2008 |
| *Leptolalax laui* Sung, Yang, & Wang, 2014 | Sung, Yang & Wang, 2014 |
| *Leptolalax liui* Fei & Ye, 1990 | Fei et al., 2009, Sung, Yang & Wang, 2014 |
| *Leptolalax maculosus* Rowley, Tran, Le, Dau, Peloso, Nguyen, Hoang, Nguyen, & Ziegler, 2016 | Rowley et al., 2016 |
| *Leptolalax maoershanensis* Yuan, Sun, Chen, Rowley, & Che, 2017 | Yuan et al., 2017 |
| *Leptolalax marmoratus* Matsui, Zainudin, & Nishikawa, 2014 | Matsui, Zainudin & Nishikawa, 2014 |
| *Leptolalax maurus* Inger, Lakim, Biun, & Yambun, 1997 | Inger et al., 1997 |
| *Leptolalax melanoleucus* Matsui, 2006 | Matsui, 2006 |
| *Leptolalax melicus* Rowley, Stuart, Neang, & Emmett, 2010 | Rowley et al., 2010b |
| *Leptolalax minimus* (Taylor, 1962) | Taylor, 1962; Ohler et al., 2011 |
| *Leptolalax nahangensis* Lathrop, Murphy, Orlov, & Ho, 1998 | Lathrop et al., 1998 |
| *Leptolalax nokrekensis* (Mathew & Sen, 2010) | Mathew & Sen, 2010 |
| *Leptolalax nyx* Ohler, Wollenberg, Grosjean, Hendrix, Vences, Ziegler, & Dubois, 2011 | Ohler et al., 2011 |
| *Leptolalax oshanensis* (Liu, 1950) | Fei et al., 2009; Fei, Ye & Jiang, 2012 |
| *Leptolalax pallidus* Rowley, Tran, Le, Dau, Peloso, Nguyen, Hoang, Nguyen, & Ziegler, 2016 | Rowley et al., 2016 |
| *Leptolalax pelodytoides* (Boulenger, 1893) | Boulenger, 1893; Ohler et al., 2011 |
| *Leptolalax petrops* Rowley, Dau, Hoang, Le, Cutajar, & Nguyen, 2017 | Rowley et al., 2017 |
| *Leptolalax pictus* Malkmus, 1992 | Malkmus, 1992 |
| *Leptolalax platycephalus* Dehling, 2012 | Dehling, 2012a |
| *Leptolalax pluvialis* Ohler, Marquis, Swan, & Grosjean, 2000 | Ohler et al., 2000; Ohler et al., 2011 |
| *Leptolalax puhoatensis* Rowley, Dau, & Cao, 2017 | Rowley, Dau & Cao, 2017 |
| *Leptolalax pyrrhops* Poyarkov, Rowley, Gogoleva, Vassilieva, Galoyan, & Orlov, 2015 | Poyarkov et al., 2015 |
| *Leptolalax sabahmontanus* Matsui, Nishikawa, & Yambun, 2014 | Matsui, Nishikawa & Yambun, 2014 |
| *Leptolalax solus* Matsui, 2006 | Matsui, 2006 |
| *Leptolalax sungi* Lathrop, Murphy, Orlov, & Ho, 1998 | Lathrop et al., 1998 |
| *Leptolalax tadungensis* Rowley, Tran, Le, Dau, Peloso, Nguyen, Hoang, Nguyen, & Ziegler, 2016 | Rowley et al., 2016 |
| *Leptolalax tamdil* Sengupta, Sailo, Lalremsanga, Das, & Das, 2010 | Sengupta et al., 2010 |
| *Leptolalax tengchongensis* Yang, Wang, Chen, & Rao, 2016 | Yang et al., 2016 |
| *Leptolalax tuberosus* Inger, Orlov, & Darevsky, 1999 | Inger, Orlov & Darevsky, 1999 |
| *Leptolalax ventripunctatus* Fei, Ye, & Li, 1990 | Fei et al., 2009; Fei, Ye & Jiang, 2012 |
| *Leptolalax zhangyapingi* Jiang, Yan, Suwannapoom, Chomdej, & Che, 2013 | Jiang et al., 2013 |

**References:**

**Anderson J. 1871.** A list of the reptilian accession to the Indian Museum, Calcutta from 1865 to 1870, with a description of some new species. *Journal of the Asiatic Society of Bengal* 40:12–39.

**Boulenger GA. 1893.** Concluding report on the reptiles and batrachians obtained in Burma by Signor L. Fea dealing with the collection made in Pegu and the Karin Hills in 1887–88. *Annali del Museo Civico di Storia Naturale di Genova* 13:304–347.

**Boulenger GA. 1900.** Descriptions of new batrachians and reptiles from the Larut Hills, Perak. *Annals and Magazine of Natural History* 6:186–194.

**Das I, Tron RKL, Rangad D, Hooroo RN. 2010.** A new species of *Leptolalax* (Anura: Megophryidae) from the sacred groves of Mawphlang, Meghalaya, north-eastern India. *Zootaxa* 2339:44–56.

**Dehling JM, Matsui M. 2013.** A new species of *Leptolalax* (Anura: Megophryidae) from Gunung Mulu National Park, Sarawak, East Malaysia (Borneo). *Zootaxa* 3670(1):33–44.

**Dehling JM. 2012a.** Eine neue Art der Gattung *Leptolalax* (Anura: Megophryidae) vom Gunung Benom, Westmalaysia/A new species of the genus *Leptolalax* (Anura: Megophryidae) from Gunung Benom, Peninsular Malaysia. *Sauria* 34:9–21.

**Dehling JM. 2012b.** Redescription of *Leptolalax gracilis* (Günther, 1872) from Borneo and taxonomic status of two populations of *Leptolalax* (Anura: Megophryidae) from Peninsular Malaysia. *Zootaxa* 3328:20–34.

**Dubois A. 1983.** Note preliminaire sur le genre *Leptolalax* Dubois, 1980 (Amphibiens, Anoures), avec diagnose d’une espece novelle du Vietnam. *Alytes* 2:147–153.

**Fei L, Hu SQ, Ye CY, Huang YZ. 2009.** *Fauna Sinica. Amphibia Vol. 2 Anura*. Science Press, Beijing, 957 pp. [In Chinese]

**Fei L, Ye CY, Jiang JP. 2012.** *Colored atlas of Chinese amphibians and their distributions*. Sichuan Publishing House of Science & Technology, Chengdu, 619 pp. [In Chinese]

**Grismer LL, Grismer JL, Youmans TM. 2004.** A new species of *Leptolalax* (Anura: Megophryidae) from Pulau Tioman, West Malaysia. *Asiatic Herpetological Research* 10:8–11.

**Günther A. 1872.** On the reptiles and amphibians of Borneo. *Proceedings of the Scientific Meetings of the Zoological Society of London* 1872:586–600.

**Humtsoe LN, Bordoloi S, Ohler A, Dubois A. 2008.** Rediscovery of a long known species, *Ixalus lateralis* Anderson, 1871. *Zootaxa* 1921:24–34.

**Inger RF, Lakim M, Biun A, Yambun P. 1997.** A new species of *Leptolalax* (Anura: Megophryidae) from Borneo. *Asiatic Herpetological Research* 7:48–50.

**Inger RF, Orlov N, Darevsky I. 1999.** Frogs of Vietnam: a report on new collections. *Fieldiana Zoology* 92:1–46.

**Inger RF, Stuebing RB, Tan FL. 1995.** New species and new records of anurans from Borneo. *Raffles Bulletin of Zoology* 43:115–132.

**Jiang K, Yan F, Suwannapoom C, Chomdej S, Che J. 2013.** A new species of the genus *Leptolalax* (Anura: Megophryidae) from northern Thailand. *Asian Herpetological Research* 4(2):100–108.

**Lathrop A, Murphy RW, Orlov N, Ho CT. 1998.** Two new species of *Leptolalax* (Anura: Megophryidae) from northern Vietnam. *Amphibia-Reptilia* 19:253–267.

**Malkmus R. 1992.** *Leptolalax pictus* sp.n. (Anura: Pelobatidae) vom Mount Kinabalu/Nord-Borneo. *Sauria* 14:3–6.

**Mathew R, Sen N. 2010 "2009".** Description of a new species of *Leptobrachium* Tschudi, 1838 (Amphibia: Anura: Megophryidae) from Meghalaya, India. *Records of the Zoological Survey of India*, 109, 91–108.

**Matsui M, Dehling JM. 2012.** Notes on an enigmatic Bornean megophryid, *Leptolalax dringi* Dubois, 1987 (Amphibia: Anura). *Zootaxa* 3317:49–58.

**Matsui M. 1997.** Call characteristics of Malaysian *Leptolalax* with a description of two new species (Anura: Pelobatidae). *Copeia*, 158–165.

**Matsui M. 2006.** Three new species of *Leptolalax* from Thailand (Amphibia, Anura, Megophryidae). *Zoological Science*, 23(9), 821–830.

**Matsui M, Belabut DM, Ahmad N, Yong HS. 2009.** A new species of *Leptolalax* (Amphibia, Anura, Megophryidae) from Peninsular Malaysia. *Zoological Science* 26(3):243–247.

**Matsui M, Nishikawa K, Yambun P. 2014.** A new *Leptolalax* from the mountains of Sabah, Borneo (Amphibia, Anura, Megophryidae). *Zootaxa* 3753(3):440–452.

**Matsui M, Zainudin R, Nishikawa K. 2014.** A New Species of *Leptolalax* from Sarawak, Western Borneo (Anura: Megophryidae). *Zoological Science* 31(11):773–779.

**Ohler A, Marquis O, Swan S, Grosjean S. 2000.** Amphibian biodiversity of Hoang Lien Nature Reserve (Lao Cai Province, northern Vietnam) with description of two new species. *Herpetozoa* 13(1/2):71–87.

**Ohler A, Wollenberg KC, Grosjean S, Hendrix R, Vences M, Ziegler T, Dubois A. 2011.** Sorting out *Lalos*: description of new species and additional taxonomic data on megophryid frogs from northern Indochina (genus *Leptolalax*, Megophryidae, Anura). *Zootaxa* 3147:1–83.

**Poyarkov NA, Rowley JJ, Gogoleva SI, Vassilieva AB, Galoyan EA, Orlov NL. 2015.** A new species of *Leptolalax* (Anura: Megophryidae) from the western Langbian Plateau, southern Vietnam. *Zootaxa* 3931(2):221–252.

**Rowley JJ, Cao TT. 2009.** A new species of *Leptolalax* (Anura: Megophryidae) from central Vietnam. *Zootaxa* 2198:51–60.

**Rowley JJ, Dau VQ, Cao TT. 2017.** A new species of *Leptolalax* (Anura: Megophryidae) from Vietnam. *Zootaxa* 4273(1):61–79.

**Rowley JJ, Dau VQ, Nguyen TT. 2013.** A new species of *Leptolalax* (Anura: Megophryidae) from the highest mountain in Indochina. *Zootaxa* 3737(4):415–428.

**Rowley JJ, Dau VQ, Hoang HD, Le DT, Cutajar TP, Nguyen TT. 2017.** A new species of *Leptolalax* (Anura: Megophryidae) from northern Vietnam. *Zootaxa* 4243(3):544–564.

**Rowley JJ, Hoang DH, Le TTD, Dau QV, Cao TT. 2010a.** A new species of *Leptolalax* (Anura: Megophryidae) from Vietnam and further information on *Leptolalax tuberosus*. *Zootaxa* 2660:33–45.

**Rowley JJ, Le DTT, Tran DTA, Hoang DH. 2011.** A new species of *Leptolalax* (Anura: Megophryidae) from southern Vietnam. *Zootaxa* 2796:15–28.

**Rowley JJ, Stuart BL, Neang T, Emmett DA. 2010b.** A new species of Leptolalax (Anura: Megophryidae) from northeastern Cambodia. *Zootaxa* 2567:57–68.

**Rowley JJ, Stuart BL, Richards SJ, Phimmachak S, Sivongxay N. 2010c**. A new species of *Leptolalax* (Anura: Megophryidae) from Laos. *Zootaxa* 2681:35–46.

**Rowley JJL, Hoang HD, Dau VQ, Le TTD, Cao TT. 2012.** A new species of *Leptolalax* (Anura: Megophryidae) from central Vietnam. *Zootaxa* 3321:56–68.

**Rowley JJL, Stuart BL, Neang T, Hoang HD, Dau VQ, Nguyen TT, Emmett DA. 2015a.** A new species of *Leptolalax* (Anura: Megophryidae) from Vietnam and Cambodia*.* *Zootaxa* 4039:401–417.

**Rowley JJL, Tran DTA, Le DTT, Dau VQ, Peloso PLV, Nguyen TQ, Hoang HD, Nguyen TT, Ziegler T. 2016.** Five new, microendemic Asian Leaf-litter Frogs (*Leptolalax*) from the southern Annamite mountains, Vietnam. *Zootaxa* 4085:63–102.

**Sengupta S, Sailo S, Lalremsanga HT, Das A, Das I. 2010.** A new species of *Leptolalax* (Anura: Megophryidae) from Mizoram, north-eastern India. *Zootaxa* 2406:56–68.

**Sung YH, Yang JH, Wang YY. 2014.** A new species of *Leptolalax* (Anura: Megophryidae) from southern China. *Asian Herpetological Research* 5(2):80–90.

**Taylor EH. 1962.** The amphibian fauna of Thailand. *University of Kansas Science Bulletin*, 43:265–599.

**Yang JH, Wang YY, Chen GL, Rao DQ. 2016.** A new species of the genus *Leptolalax* (Anura: Mehophryidae) from Mt. Gaoligongshan of Western Yunnan Province, China. *Zootaxa* 4088(3):379–394.

**Yuan ZY, Sun R, Chen J, Rowley JJL, Wu Z, Hou S, Wang S, Che J. 2017.** A new species of the genus *Leptolalax*(Anura: Megophryidae) from Guangxi, China. *Zootaxa* 4300:551–570.
