# Supplementary material for: Description of two new sympatric species of the genus Leptolalax (Anura: Megophryidae) from western Yunnan of China
Source: PeerJ. 2018 Apr 10;6:e4586. doi: 10.7717/peerj.4586 (PMC5898428; doi:10.7717/peerj.4586)
Supplement: Appendix S2 [file peerj-06-4586-s003.docx]

**Appendix 2:** Specimens of *Leptolalax* examined in this study.

Institutional abbreviations used are **CIB** = Chengdu Institute of Biology, the Chinese Academy of Sciences, Chengdu; **SYS** = The Museum of Biology, Sun Yat-sen University, Guangzhou; **KFBG** = Herpetology collection of Kadoorie Farm and Botanic Garden, Hong Kong.

*Leptolalax alpinus*: China, Yunnan Province, Mt. Wuliangshan: CIB 24353 (Holotype), CIB 24354; SYS a 003915–3917, 003927.

*Leptolalax laui*: China, Hong Kong: SYS a002057 (Holotype), SYS a002058; Guangdong Province, Shenzhen: SYSa 001505–001507, 001515–001521.

*Leptolalax liui*: China, Fujian Province, Mt. Wuyishan: CIB 24355 (Holotype), CIB 24356, SYS a001571–001578, 001595– 001599.

*Leptolalax tengchongensis*: China, Yunnan Province, Tengchong: SYS a004600 (Holotype), SYS a004596–SYS a004599, SYS a004601–4602.

*Leptolalax ventripuntactus*: China, Yunnan Province, Yingjiang: KFBG 14423, 14509–14513, 14531.
